# Assessing causal links between age at menarche and adolescent mental health: a Mendelian randomisation study

**DOI:** 10.1186/s12916-024-03361-8

**Published:** 2024-04-12

**Authors:** Adrian Dahl Askelund, Robyn E. Wootton, Fartein A. Torvik, Rebecca B. Lawn, Helga Ask, Elizabeth C. Corfield, Maria C. Magnus, Ted Reichborn-Kjennerud, Per M. Magnus, Ole A. Andreassen, Camilla Stoltenberg, George Davey Smith, Neil M. Davies, Alexandra Havdahl, Laurie J. Hannigan

**Affiliations:** 1 Department of Psychology, University of Oslo, Oslo, Norway; 2 Nic Waals Institute, Lovisenberg Diaconal Hospital, Oslo, Norway; 3 PsychGen Centre for Genetic Epidemiology and Mental Health, Norwegian Institute of Public Health, Oslo, Norway; 4 MRC Integrative Epidemiology Unit, Bristol Medical School, University of Bristol, Bristol, UK; 5 School of Psychological Science, University of Bristol, Bristol, UK; 6 Centre for Fertility and Health, Norwegian Institute of Public Health, Oslo, Norway; 7 Department of Epidemiology, Harvard T.H. Chan School of Public Health, Boston, USA; 8 Promenta Research Center, Department of Psychology, University of Oslo, Oslo, Norway; 9 Institute of Clinical Medicine, University of Oslo, Oslo, Norway; 10 NORMENT Centre, Institute of Clinical Medicine, University of Oslo, Oslo, Norway; 11 Division of Mental Health and Addiction, Oslo University Hospital, Oslo, Norway; 12 Department of Global Public Health and Primary Care, University of Bergen, Bergen, Norway; 13 NORCE Norwegian Research Centre, Bergen, Norway; 14 Division of Psychiatry, University College London, London, UK; 15 Department of Statistical Sciences, University College London, London, UK; 16 KG Jebsen Center for Genetic Epidemiology, Department of Public Health and Nursing, Norwegian University of Science and Technology, Trondheim, Norway

**Keywords:** Depression, Age at menarche, Mendelian randomisation, MoBa, MBRN

## Abstract

**Background:**

The timing of puberty may have an important impact on adolescent mental health. In particular, earlier age at menarche has been associated with elevated rates of depression in adolescents. Previous research suggests that this relationship may be causal, but replication and an investigation of whether this effect extends to other mental health domains is warranted.

**Methods:**

In this Registered Report, we triangulated evidence from different causal inference methods using a new wave of data (*N* = 13,398) from the Norwegian Mother, Father, and Child Cohort Study. We combined multiple regression, one- and two-sample Mendelian randomisation (MR), and negative control analyses (using pre-pubertal symptoms as outcomes) to assess the causal links between age at menarche and different domains of adolescent mental health.

**Results:**

Our results supported the hypothesis that earlier age at menarche is associated with elevated depressive symptoms in early adolescence based on multiple regression (*β* =  − 0.11, 95% CI [− 0.12, − 0.09], *p*_one-tailed_ < 0.01). One-sample MR analyses suggested that this relationship may be causal (*β* =  − 0.07, 95% CI [− 0.13, 0.00], *p*_one-tailed_ = 0.03), but the effect was small, corresponding to just a 0.06 standard deviation increase in depressive symptoms with each earlier year of menarche. There was also some evidence of a causal relationship with depression diagnoses during adolescence based on one-sample MR (OR = 0.74, 95% CI [0.54, 1.01], *p*_one-tailed_ = 0.03), corresponding to a 29% increase in the odds of receiving a depression diagnosis with each earlier year of menarche. Negative control and two-sample MR sensitivity analyses were broadly consistent with this pattern of results. Multivariable MR analyses accounting for the genetic overlap between age at menarche and childhood body size provided some evidence of confounding. Meanwhile, we found little consistent evidence of effects on other domains of mental health after accounting for co-occurring depression and other confounding.

**Conclusions:**

We found evidence that age at menarche affected diagnoses of adolescent depression, but not other domains of mental health. Our findings suggest that earlier age at menarche is linked to problems in specific domains rather than adolescent mental health in general.

**Supplementary Information:**

The online version contains supplementary material available at 10.1186/s12916-024-03361-8.

## Background

Early pubertal timing has been associated with problems in a wide range of adolescent mental health domains (e.g., depression [1–13], anxiety [7, 14, 15], conduct disorders [3, 7, 16–19], and attention-deficit hyperactivity disorder (ADHD) [6]) across different indicators of pubertal development and across sexes [20]. The consistency of associations between early timing and adolescent mental health has led to the hypothesis that early pubertal timing is a transdiagnostic risk factor for psychopathology in adolescents [21].

Despite the apparent generality of associations between early pubertal timing and adolescent mental health, the prominent rise in rates of female depression beginning during puberty [22] has led to this outcome receiving particular empirical focus [23]. The timing of puberty in females is commonly indexed using the onset of menses (menarche). Earlier age at menarche has been associated with elevated depressive symptoms in adolescents in several observational studies [4, 24–32], but not in all [33–37], and also higher rates of clinical depression during adolescence [31, 37]. However, although early pubertal maturation in females has been associated with a wide range of problems in adolescence, these associations may dissipate by adulthood [3, 38]. A notable exception in a large prospective study was that heightened risk of depression persisted into young adulthood for early maturers, in particular, for those with a history of conduct disorder [3].

The association between pubertal timing and depression in adolescent females may be due to the biological underpinnings of reproductive maturation. The female sex hormone estradiol increases with puberty and is associated with depression [39, 40], and hormonal contraceptive use has been associated with higher levels of depressive symptoms, especially in adolescence [41]. In fact, it has been found that the stage of breast development (governed primarily by estradiol) was associated with depression independently of the timing of menarche in adolescent females from the Avon Longitudinal Study of Parents and Children (ALSPAC) [42]. Interestingly, a recent study in the same sample found that a polygenic score for age at menarche showed a potential indirect association with adolescent depressive symptoms through the stage of breast development [43]. Alongside psychosocial pathways (i.e., visible breast development leading to unwanted sexual attention at a younger age), increases in estradiol represent a plausible biological mechanism for the link between early pubertal timing and depression in females.

Despite the series of observational studies, it is unknown whether the link between age at menarche and depression represents a truly causal relationship. This is important because several robust observational associations in epidemiology have turned out not to be causal and may instead have been the result of confounding (i.e., vitamin E supplement use and cardiovascular disease [44]). In the case of associations between age at menarche and depression, body mass index (BMI) is a particularly likely candidate for confounding given the robust (and plausibly causal) links between BMI and age at menarche [45] and between BMI and depression [46–48]. Failure to appropriately account for potential confounding, especially by BMI [4, 25, 27–30, 32–37], has been a relatively common shortcoming of the literature on this topic to date. In previous studies that explicitly controlled for BMI, the relationship was somewhat attenuated [26, 49]. Another study found BMI to be a partial mediator of the relationship between earlier menarche and depression [31].

Mendelian randomisation (MR) is a causal inference method that can be implemented in instrumental variable analyses [50, 51], which is particularly useful when experimental manipulation of the variable of interest is not ethical or feasible. Since hundreds of genetic variants are strongly linked with age at menarche [52], single nucleotide polymorphisms (SNPs) that are independently associated with this phenotype can be used as genetic instruments in MR analyses. The logic of MR is analogous to that of a randomised controlled trial (RCT). Unlike in an RCT design where individuals are randomly assigned to experimental groups, in MR, we use random “assignment” to genotype (ensured by the random transmission of one of two possible alleles at each genetic locus from each parent to their child at conception [53]). Specifically, these genetic variants are used as instrumental variables, serving as a genetic proxy for age at menarche.

Whereas self-reported age at menarche may be associated with several different confounders (even if precisely measured), the genetic instrument is assumed to be independent of such confounding. Both the widespread genetic influence on age at menarche [52] and the high accuracy and reliability of self-reported age at menarche [54] jointly increase the strength of the genetic instrument employed here, which serves to improve study power and minimise weak instrument bias [55]. The strength of the genetic instrument makes MR especially valuable for advancing menarche research. Provided that some important assumptions of MR hold true, we can estimate the causal effects of age at menarche on adolescent mental health.

A previous study found preliminary evidence that the relationship between age at menarche and depression in early adolescence may be causal, using MR in ALSPAC (*N* = 2404) [56]. Specifically, they found that early age at menarche resulted in more depressive symptoms at age 14 (independent of BMI), but not later in adolescence. However, this study had low power due to a modest sample size for MR. Here, we aim to replicate the 14-year analyses in adolescents from a larger birth cohort, the Norwegian Mother, Father, and Child Cohort Study (MoBa) [57]. This replication will allow for a confirmatory and higher-powered test of the hypothesis that earlier age at menarche is causally related to adolescent depression.

Beyond replicating its key finding, we will also extend the previous approach [56] in several key ways. First, we will test whether the effects of earlier age at menarche extend to other domains of mental health (anxiety disorders, conduct disorder (CD), oppositional defiant disorder (ODD), and ADHD), independent of associations with depression. Second, we will use multivariable methods to examine different confounders or mechanisms, by simultaneously including genetic instruments for childhood body size, adult BMI, or estradiol in the MR model together with age at menarche. Third, in line with recommendations to triangulate evidence across approaches for robust causal inference [58], we will combine MR with negative control analyses using symptoms prior to puberty as a negative control outcome. This triangulation is particularly important in the context of replication studies, given that the same sources of bias could lead to results being replicated in another study using the same methodology [59, 60].

A previous hypothesis-free MR phenome-wide association study identified potential causal effects of age at menarche on adult mental health [61], but these were not followed up with replication in any independent cohorts. Here, we take a confirmatory approach, testing causal hypotheses about the role of age at menarche in the aetiology of developing mental health disorders. This is important in part because a causal effect of age at menarche may help explain the sharp rise in depression rates among females from early adolescence [22]. This research might further help with identifying female adolescents at increased risk, facilitating early identification and prevention of mental health problems in adolescence and beyond.

To test our hypotheses, we make use of the Registered Report format, demonstrating its applicability to epidemiological analyses of cohort data when a new wave of data collection ensures that the exposure and outcome data have not been observed prior to the analytic choices being made. This format, combined with several sensitivity tests, will strengthen our statistical inferences by preserving false-positive rates at the specified level [62] and ultimately increase confidence in the causal conclusions that are drawn.

We addressed the following research questions: (1) To what extent is age at menarche associated with adolescent depression? (2) Does age at menarche associate with symptoms or diagnoses in other domains of mental health, independent of depression? (3) What is the evidence for a causal link between age at menarche and depression? and (4) Is there evidence of causal links between age at menarche and other domains of mental health? The specific hypotheses for each research question are listed in Table 1.

**Table 1:** Pre-specified research questions and hypotheses in this Registered Report

Research questions	Hypotheses
To what extent is age at menarche associated with adolescent depression?	We hypothesised that earlier age at menarche would be associated with elevated depressive symptoms at age 14 (H1a)
	We hypothesised that earlier age at menarche would be associated with higher rates of depression diagnoses during adolescence (H1b)
Does age at menarche associate with symptoms or diagnoses in other domains of mental health (anxiety, CD, ODD, or ADHD), independent of depression?	We hypothesised that age at menarche would be associated with symptoms in other domains at age 14, independent of depressive symptoms (H2.1–4a)
	We hypothesised that age at menarche would be associated with diagnoses in other domains in adolescence, independent of depressive disorders (H2.1–3b)
What is the evidence for a causal link between age at menarche and depression?	We hypothesised that earlier age at menarche would show a causal relationship, resulting in elevated depressive symptoms at age 14 (H3a)
	We hypothesised that earlier age at menarche would show a causal relationship, resulting in higher rates of depression diagnoses during adolescence (H3b)
Is there evidence of causal links between age at menarche and other domains of mental health?	We hypothesised that age at menarche would show a causal relationship with symptoms in other domains (H4.1–4a)
	We hypothesised that age at menarche would show a causal relationship with rates of diagnoses in other domains (H4.1–3b)

## Methods

### Design

#### Sample

The Norwegian Mother, Father, and Child Cohort Study (MoBa) is a population-based pregnancy cohort study conducted by the Norwegian Institute of Public Health [57]. Pregnant women and their partners were recruited from all over Norway at approximately 17 weeks gestation between 1999 and 2008. The women consented to participation in 41% of the pregnancies. The cohort includes approximately 114,500 children, 95,200 mothers, and 75,200 fathers. The current study is based on version 12 of the quality-assured data files released for research in January 2019. We also used data from the Medical Birth Registry of Norway (MBRN).

**Fig. 1:**
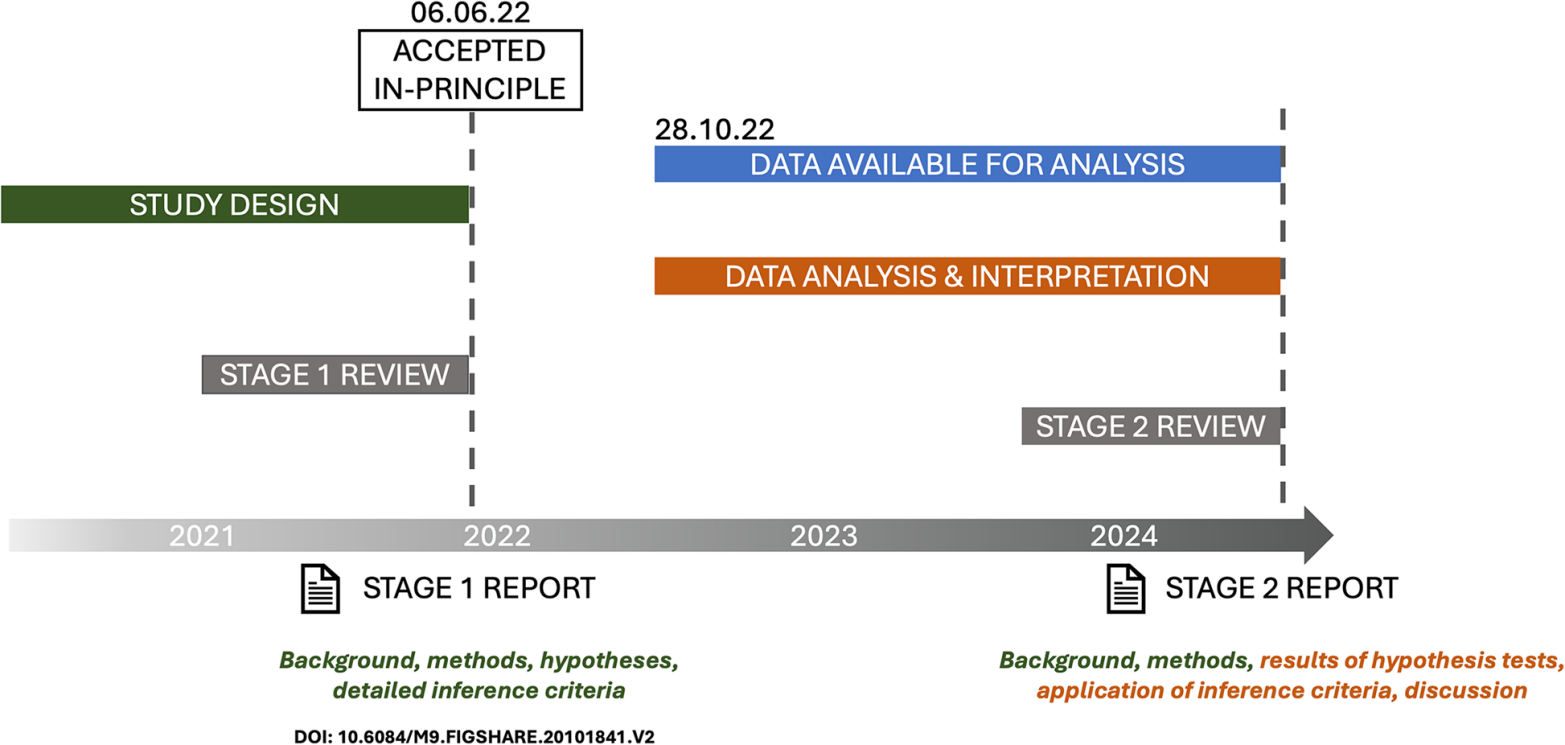
Overview of the Registered Report process

In MoBa, phenotype data have been collected by questionnaires from early pregnancy to middle childhood, provided primarily by mothers (around weeks 17, 22, and 30 of pregnancy and at child ages 0.5, 1.5, 3, 5, and 8 years). This project also made use of an ongoing wave of data collection in adolescence (questionnaires returned at ~ 14.4 years; hereafter age 14). The 14-year data were not available to us during the preparation of the stage 1 element of the Registered Report (see Fig. 1).

#### Inclusion criteria and sample size

We included all MoBa females (as registered at birth in MBRN) with any available phenotype data at age 14. There were 13,398 females with 14-year data, of which 9832 were genotyped.

#### Measures

##### Exposures

Self-reported age at menarche (in years) from the 14-year questionnaire was included as the main exposure. We ran the observational analyses with both a continuous and a categorical (early/average/late) variable based on reported age at menarche (see Additional file 1 for further details). Values were imputed for those who had not yet reached menarche at age 14, using information about the stage of pubertal development, as well as all the covariates and outcomes (see Additional file 1). We also included the self-reported stage of breast development at age 14 as an additional exposure for sensitivity analyses.

##### Mental health problems

Depressive symptoms were assessed through the Short Mood and Feelings Questionnaire (SMFQ; 13 items) [63]. We also computed a dichotomised version of the SMFQ (see Additional file 1). Anxiety symptoms were assessed through a short form of the Screen for Child Anxiety-Related Disorders (SCARED; 5 items) [64]. Behaviour problems (CD, ODD, and ADHD) were assessed with the Rating Scale for Disruptive Behaviour Disorders (RS-DBD; 34 items) [65]. All symptom outcomes were log or square root transformed due to non-normal distributions. The measures were treated as continuous, and scores were standardised to have a mean of 0 and a standard deviation of 1. Information about the psychometric properties of the scales is provided in Additional file 2: Table S1. An overview of all variables included in the study and their processing is in Additional file 3: Table S2.

##### Psychiatric diagnoses

We linked to the “control and payment of health refunds” database (KUHR) and the Norwegian Patient Registry (NPR) to obtain psychiatric diagnoses from medical records (see Additional file 1 for diagnostic codes and further details). Individuals were classified as a “case” in the case–control analysis if they had received a relevant diagnosis in either primary (covered by KUHR) or secondary health care (covered by NPR) during adolescence (between ages 10 and 17).

##### Covariates

We included BMI at ages 8 and 14, age at questionnaire return, maternal and paternal age, parental education and income, financial problems, parental cohabitation, parity, and maternal prenatal and postnatal depression as covariates (see Additional file 3: Table S2).

##### Genotyping and quality control

In MoBa, blood samples were obtained from children (umbilical cord) at birth [66]. The genotyping and quality control have been described in detail elsewhere [67].

##### Genetic instruments for Mendelian randomisation

A recent genome-wide association study (GWAS) meta-analysis of 42 studies involving 329,345 post-pubertal women of European ancestry found 389 independent signals associated with self-reported age at menarche, reaching the conventional threshold for genome-wide significance (*p* < 5 × 10^−8^) in the discovery sample [52]. These variants were largely replicated in a sample of 39,543 post-pubertal women from the Icelandic deCODE study, explaining 7.4% of the variance in age at menarche. First, we subset these genome-wide significant variants to single nucleotide polymorphisms (SNPs) only by removing insertions and deletions. Then, we extracted these SNPs (as available) from the genetic data in MoBa, which did not contribute to the GWAS meta-analysis. Having subset to genome-wide significant SNPs available in the MoBa cohort, we then clumped them for independence (linkage disequilibrium *R*^2^ = 0.001, clumping window = 10,000 kb). For the one-sample MR, we used this set of SNPs to construct a weighted genetic risk score based on published GWAS effect estimates. The score was computed as the weighted sum of the age-at-menarche-increasing alleles across the selected SNPs. Specifically, we multiplied the number of effect alleles (0, 1, or 2; or if imputed, probabilities of effect alleles) at each SNP by their weight (GWAS SNP-trait association), then summed and divided by the total number of SNPs used. Genotyping batch and the first 20 principal components were regressed out of the genetic instruments, the latter to control for confounding by population stratification.

We also prepared the age at menarche summary statistics—along with summary statistics for estradiol [68], adult BMI [69], recalled childhood body size [69], major depression [70], and the 14-year symptom outcomes in MoBa—for use in two-sample MR analyses (see Additional file 4 for details [69–74]). We also employed Steiger filtering [75] to create another genetic instrument for age at menarche, excluding SNPs that were more predictive of depression at age 14 than age at menarche. This primarily served to prevent reverse causation and to remove potential pleiotropic pathways other than the causal pathway of interest.

### Statistical analysis

#### Observational analyses

First, we ran linear regression analyses to estimate the observational associations between age at menarche and continuous symptom outcomes, accounting for the effects of covariates. In addition, we ran logistic regression analyses to estimate observational associations with the diagnostic outcomes from registry data, accounting for the effects of covariates. All models were run with and without the covariates described above to obtain adjusted and unadjusted estimates, and inferences were based on the adjusted estimates.

#### Mendelian randomisation analyses

**Fig. 2:**
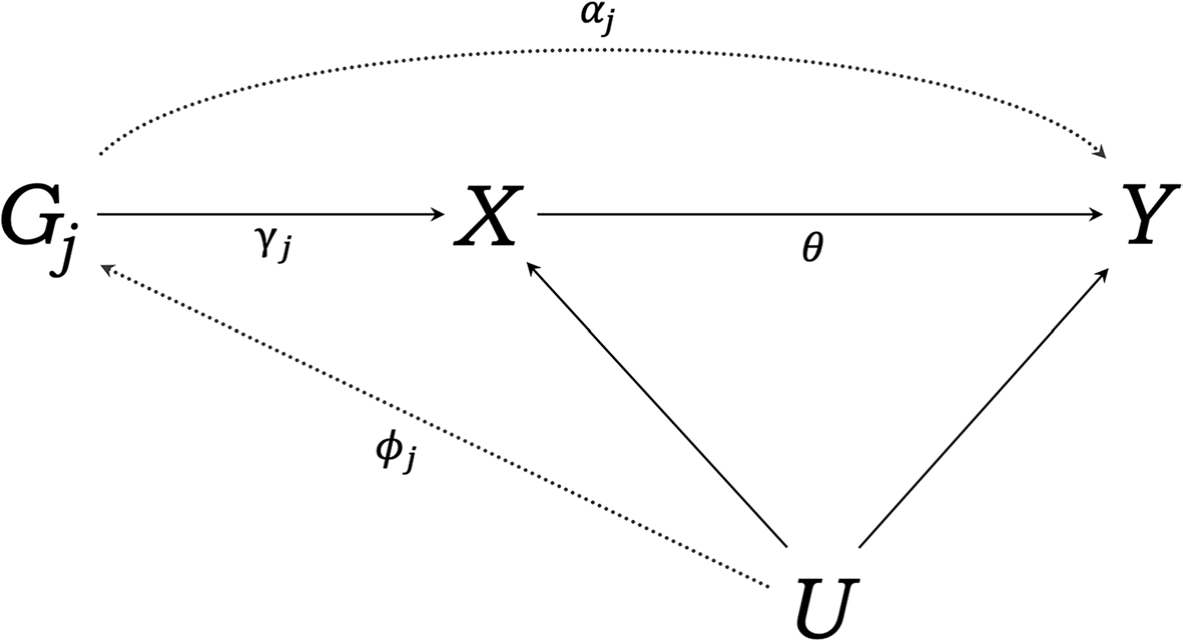
Directed acyclic graph illustrating the Mendelian randomisation design. *G*_*j*_ is the *j*th genetic variant, with direct effect *γ*_*j*_ on exposure *X*, and direct effect *α*_*j*_ on outcome *Y*; *θ* is the estimated causal effect of the exposure on the outcome; *ϕ*_*j*_ is the relationship between confounders *U* and *G*_*j*_; dotted lines represent possible violations of the MR assumptions

To avoid problems related to confounding and reverse causation common to traditional observational methods, MR uses *j* genetic variants *G*_*1*_,* G*_*2*_, …, *G*_*j*_ as a proxy for the exposure *X* to estimate the association between the exposure *X* and the outcome *Y* (see Fig. 2 for an illustrative diagram) [50]. The obtained estimate is assumed to be independent of potential confounders *U*. This assumption builds on Mendel’s first and second law of inheritance [53]. The two laws are (1) the segregation of alleles at the same locus is independent (equal segregation) and (2) the alleles of different genes are inherited independently of each other during gamete formation (independent assortment).

##### MR assumptions

The three main assumptions of MR are (1) that the instrument *G*_*j*_ is associated with the exposure *X*, called the relevance assumption; (2) that there are no unmeasured confounders of the gene-outcome association *U*, called the independence assumption; and (3) that the genetic variants *G*_*j*_ affect the outcome *Y* only through the exposure *X*, called exclusion restriction. While assumption 1 can be verified empirically, assumptions 2 and 3 are empirically unverifiable (but potentially falsifiable). Owing to how instrumental variable analyses are estimated, violations of these assumptions may lead to strong biases in the estimates; therefore, such estimates should be interpreted with care and in conjunction with other evidence [76]. Several sensitivity analyses that have been developed to address potential bias from violations of the MR assumptions were employed here [51].

##### One-sample MR

In the one-sample MR analyses of continuous symptom variables, we used two-stage least squares (2SLS) regression. In the 2SLS approach, self-reported age at menarche *X* was first regressed on the genetic variants *G*_*j*_, obtaining the predicted values. In the second stage, the regression of the outcome *Y* on the exposure *X* is estimated as usual, replacing self-reported age at menarche with the predicted values from the first stage, hereafter referred to as “genetically predicted age at menarche”. For binary outcomes, a logistic model was used in the second stage. We applied a post-estimation correction of the standard errors (the *HC1* option in the *sandwich* R package [77]) for both continuous and binary outcomes. In addition to one-sample MR, we conducted two-sample MR analyses based on GWAS of the symptom outcomes in MoBa. Combining one- and two-sample MR is beneficial even when the same outcome sample is used, since any bias from weak instruments would skew the one-sample estimate towards the (confounded) observational estimate and the two-sample estimate towards the null [78]. In addition, conducting two-sample MR maximises the availability of sensitivity analyses to test the MR assumptions.

##### Two-sample MR

In the two-sample MR analyses, only the genotype-outcome (*G-Y*) association was estimated in MoBa. For these analyses (using the *TwoSampleMR* package [71]), we extracted estimates for the genotype-exposure (*G-X*) association from summary-level data from the age-at-menarche GWAS [52] and produced a set of SNP-specific Wald estimates by calculating the ratio between the *G-X* and the *G-Y* associations. We tested the heterogeneity between the Wald ratios using SNP-specific *Q* statistics. Then, these estimates were combined using the inverse variance weighted (IVW) meta-analysis approach to obtain an estimate of the causal effect.

##### Multivariable MR and MR sensitivity analyses

We conducted a battery of sensitivity analyses across the one- and two-sample MR (described in Additional file 5 [79–85]) to assess the robustness of results and the impact of horizontal pleiotropy—where the genetic variants affect the outcome through other pathways than the exposure of interest. A likely source of horizontal pleiotropy is via BMI, which is therefore included as an additional exposure in multivariable Mendelian randomisation (MVMR) analyses [83]. The employed two-sample MR sensitivity analyses (MR-Egger [79], MR pleiotropy residual sum and outlier (MR-PRESSO) [80], weighted median [81], and contamination mixture methods [82]) make different assumptions about horizontal pleiotropy, and we consider effects that are consistent across these approaches to be more likely causal, in line with a triangulation approach.

#### Equivalence testing

We additionally used equivalence testing to assess whether estimated effects could be considered practically equivalent to 0. The region of practical equivalence to 0 was set, for each analysis, by pre-defining the smallest effect size of interest (SESOI). Equivalence tests were carried out with a 5% alpha. Details of the full procedure for setting the SESOI and carrying out the equivalence testing are described in Additional file 6 [20, 24, 29, 31, 38, 42, 49, 56, 86–90].

#### Negative control analyses

Since an individual’s age at menarche cannot directly influence their mental health prior to puberty, childhood symptoms can serve as a negative control outcome in our study. Such analyses can be used to detect unmeasured confounding in the context of MR, given that the negative control outcome is associated with confounders in a similar way to the outcome of interest [87]. Here, we estimate the causal effect of age at menarche on symptoms of depression, anxiety CD, ODD, and ADHD measured before puberty (at 8 years). We formally compare the estimate from the main analysis with this negative control by testing whether the 14-year estimate is consistent with an effect more extreme than the lower 95% CI of the 8-year estimate.

#### Missingness/handling of missing data

Within the 14-year sample, we used multiple imputation (MI) to account for missing data in all variables (see Additional file 3 for the amount of missing data per variable). Importantly, some individuals reported having not yet had their first menstrual period in the 14-year questionnaire. Imputed values for age at menarche for these individuals were not allowed to be lower than 15 years. Further details on the MI and handling of outliers are presented in Additional file 1. In addition to MI, we used inverse probability weighting to address potential bias from selective attrition out of the study over time (see Additional file 1).

#### Power calculations

For the stage 1 submission, power analyses were conducted in R by simulation for all null hypothesis significance tests (NHSTs) and equivalence tests used to investigate each hypothesis (see summary in Table 2, and further details in Additional file 7).

**Table 2:** Design table with a summary of questions, hypotheses, power, analyses, and interpretation

Question	Hypothesis	Power analysis	Analysis plan	Interpretation of different outcomes
To what extent is age at menarche associated with adolescent depression?	H1a) We hypothesised that an earlier age at menarche would be associated with elevated depressive symptoms at age 14	Power was calculated in R based on simulated data (1000 replicates). For this analysis, we simulated a depressive symptoms variable (*M* = 5.71, SD = 4.93; values taken from Sequeira et al.). Results indicated 95% power to detect an effect of Cohen’s *D* ≥ 0.08 at *N* = 12,000 and ≥ 0.06 at *N* = 13,000 for the association between age at menarche and depressive symptoms	Linear regression models with age of menarche as the independent variable and depressive symptoms at age 14 as the dependent variable For this analysis, we also added depressive symptoms at age 8 (“pre-pubertal symptoms”) as a covariate Perform a test of inferiority for the estimate against *D* = 0.23, the smallest effect size of interest (SESOI)	A negative and statistically significant association between age at menarche and depressive symptoms at age 14 would suggest that adolescent girls with earlier age at menarche show elevated depressive symptoms. A non-significant association would suggest an absence of evidence for this If the inferiority test reveals that our estimate is consistent with effects more extreme than the SESOI, this would suggest that the association is of a meaningful magnitude Hypothesis 1a would be supported if (1) the coefficient for the effect of age at menarche on 14-year depressive symptoms was significantly less than 0 (one-tailed test; alpha 5%) in the pre-pubertal symptoms-adjusted model and (2) we failed to reject the null hypothesis that this effect in the population was at least as large as the SESOI (one-tailed test; alpha 5%) If we can reject an effect size at least as large as the SESOI, then the effect would be declared practically equivalent to 0, irrespective of the result of the NHST We would explicitly remain undecided on the hypothesis if we could not reject an effect of exactly 0 (in the NHST) or an effect at least as large as the SESOI. Both these conditions apply to all remaining hypotheses
To what extent is age at menarche associated with adolescent depression?	H1b) We hypothesised that an earlier age at menarche would be associated with higher rates of depression diagnoses during adolescence	For this power analysis, we simulated a binary depression diagnosis variable, based on a pre-specified prevalence and association with age at menarche (varied across scenarios at 2%, 6%, 10%, 14%; *D* = 0 to − 0.26 in increments of 0.02). Results indicated 95% power to detect an effect of Cohen’s *D* ≥ 0.14 at both *N* = 12,000 and *N* = 13,000 for depression prevalence of 6% or higher	Logistic regression models with covariates and age of menarche as independent variables and depression diagnoses during adolescence (ages 10–17) as the dependent variable For this analysis, we would also add depression diagnoses during childhood (ages 0–8; “pre-pubertal depression status”) as a covariate Perform a test of inferiority for the estimate against *D* = 0.23, the smallest effect size of interest (SESOI)	A negative and statistically significant association between age at menarche and depression diagnoses during adolescence would suggest that adolescent girls with earlier age at menarche show higher rates of depression. A non-significant association would suggest an absence of evidence for this If the inferiority test reveals that our estimate is consistent with effects more extreme than the SESOI, this would suggest that the association is of a meaningful magnitude Hypothesis 1b would be supported if (1) the coefficient for the effect of age at menarche on odds of depression diagnoses was significantly less than 0 (one-tailed test; alpha 5%) and (2) we failed to reject the null hypothesis that this effect in the population was at least as large as the SESOI (one-tailed test; alpha 5%)
Does age at menarche associate with symptoms or diagnoses in other domains of mental health, independent of depression?	H2.1–4a) We hypothesised that age at menarche would be associated with symptoms of anxiety (H2.1a), CD (H2.2a), ODD (H2.3a), and ADHD (H2.4a) at age 14, independent of depressive symptoms	For this power analysis, we simulated anxiety, CD, ODD, and ADHD symptoms, with distributions based on the same variables in MoBa data at 8 years. Results indicated 95% power to detect an effect of Cohen’s *D* ≥ 0.12 at both simulated sample sizes (*N* = 12,000 and *N* = 13,000)	Separate multiple linear regression models with covariates and self-reported age at menarche included as independent variables, and either symptoms of anxiety, CD, ODD, or ADHD as the dependent variable Add depressive symptoms as a covariate in each model to examine whether any associations are independent of co-occurring depressive symptoms Then, add a measure of each domain at age 8 as a covariate in the age 14 model for that domain, to examine whether associations are independent of pre-pubertal symptoms (“fully adjusted” models) Perform a test of equivalence for the estimate against *D* = − 0.22–0.22, the equivalence bounds based on our smallest effect size of interest (SESOI)	Any association between age at menarche and anxiety, CD, ODD, or ADHD that significantly differed from 0 at age 14 would suggest that there was an association with age at menarche in that domain. No significant associations would suggest an absence of evidence for this If the equivalence test reveals that our estimate is consistent with effects outside of the region of practical equivalence, this would suggest that the association is of a meaningful magnitude Each of the hypotheses 2.1–4a would be supported if (1) the coefficient for the association between age at menarche and that domain of 14-year symptoms in the fully adjusted model was different from 0 (two-tailed tests, 5% alpha) and (2) we failed to reject the null hypothesis that the association in the population was at least as extreme as the SESOI in either direction (two one-tailed tests, 5% alpha)
Does age at menarche associate with symptoms or diagnoses in other domains of mental health, independent of depression?	H2.1–3b) We hypothesised that age at menarche would be associated with diagnoses in other domains: anxiety disorders (H2.1b), conduct disorders (H2.2b) including CD and ODD, and ADHD (H2.3b) during adolescence, independent of depressive disorders	This power analysis was similar to H2.1–4a above, apart from different equivalence bounds and the use of two-tailed tests. Results indicated 95% power to detect an association of Cohen’s *D* ≥ 0.20 at *N* = 12,000 and ≥ 0.18 at *N* = 13,000 between age at menarche and diagnoses of anxiety, ADHD, and conduct disorders (CD and ODD), for diagnosis prevalence of 2% or higher	Separate multivariate logistic regression models with covariates and self-reported age at menarche included as independent variables and either diagnoses of anxiety, ADHD, or conduct disorders as the dependent variable Add depression diagnostic status (ages 10–17) as a covariate in each model to examine whether any associations are independent of comorbid depression Then, add pre-pubertal diagnostic status (ages 0–8) as a covariate in the age 14 model for that domain, to examine whether associations are independent of prior diagnoses (“fully adjusted” models) Perform a test of equivalence for the estimate against *D* = − 0.22–0.22, the equivalence bounds based on our smallest effect size of interest (SESOI)	Any association between age at menarche and anxiety disorders, ADHD, or conduct disorders would suggest that there was an association with age at menarche in that domain. No significant associations would suggest an absence of evidence for this If the equivalence test reveals that our estimate is consistent with effects outside of the region of practical equivalence, this would suggest that the association is of a meaningful magnitude Each of the hypotheses 2.1–3b would be supported if (1) the coefficient for the association between age at menarche and diagnoses in that domain in the fully adjusted model was different from 0 (two-tailed tests, 5% alpha) and (2) we failed to reject the null hypothesis that the association in the population was at least as extreme as the SESOI in either direction (two one-tailed tests, 5% alpha)
What is the evidence for a causal link between age at menarche and depression?	H3a) We hypothesised that an earlier age at menarche would result in elevated depressive symptoms at age 14	Power was calculated for simulated genetic instruments with an *R*^2^ for the instrument-exposure association of 0.05, 0.075, and 0.1. Results indicated 95% power to detect an average causal effect (using two-stage least squares regression; 2SLS) of Cohen’s *D* ≥ 0.2 when the *R*^2^ of the instrument is 0.075 or above at either simulated sample size (*N* = 9500 and *N* = 10,500)	2SLS regression with the genetic risk score for age at menarche as the instrument, reported age at menarche as the exposure, and depressive symptoms at age 14 as the outcome 2SLS regression with depressive symptoms at age 8 as the outcome, as a negative control outcome Perform a test of inferiority for the estimate against *D* = 0.25, the smallest effect size of interest (SESOI) Two-sample multivariable MR with genetic instruments for age at menarche and childhood body size or adult body mass index as instruments, to estimate the direct effect of age at menarche on depressive symptoms Two-sample MR sensitivity analyses: MR-Egger, MR-PRESSO, weighted median, and contamination mixture	In the 2SLS regression, a negative and statistically significant estimate of the link between genetically predicted age at menarche and depressive symptoms at age 14 would suggest that earlier age at menarche was causally linked with adolescent depressive symptoms. A non-significant association would suggest an absence of evidence for this If the inferiority test reveals that our estimate is consistent with effects more extreme than the SESOI, this would suggest that the association is of a meaningful magnitude Hypothesis 3a would be supported if (1) the coefficient for the causal effect of age at menarche on 14-year depressive symptoms was significantly less than 0 (one-tailed test; alpha 5%), (2) we failed to reject the null hypothesis that this causal effect in the population was at least as large as the SESOI (one-tailed test; alpha 5%), and (3) we failed to reject the null hypothesis that this causal effect in the population was at least as large as the upper bound of the negative control estimate (one-tailed test; alpha 5%) If the hypothesis was supported, consistent results across the two-sample MR sensitivity analyses would provide further evidence of causality
What is the evidence for a causal link between age at menarche and depression?	H3b) We hypothesised that an earlier age at menarche would result in higher rates of depression diagnoses during adolescence	Power was calculated for simulated genetic instruments with an *R*^2^ for the instrument-exposure association of 0.05, 0.075, and 0.1. Results indicated 95% power to detect a causal effect (using logistic 2SLS) of Cohen’s *D* ≥ 0.24 when the *R*^2^ of the instrument is ≥ 0.075 and depression prevalence ≥ 10% at either simulated sample size (*N* = 9500 and *N* = 10,500)	Multivariate logistic (two-stage) regression with the genetic risk score for age at menarche as the instrument, self-reported age at menarche as the exposure, and depression diagnoses during adolescence (ages 10–17) as the outcome Perform a test of inferiority for the estimate against *D* = 0.25, the smallest effect size of interest (SESOI)	In the 2SLS regression, a negative and statistically significant association between genetically predicted age at menarche and depression diagnoses during adolescence would suggest that earlier age at menarche caused higher rates of adolescent depression. A non-significant association would suggest an absence of evidence for this If the inferiority test reveals that our estimate is consistent with effects more extreme than the SESOI, this would suggest that the association is of a meaningful magnitude Hypothesis 3b would be supported if (1) the coefficient for the causal effect of age at menarche on depressive disorders was significantly less than 0 (one-tailed test; alpha 5%) and (2) we failed to reject the null hypothesis that this causal effect in the population was at least as large as the SESOI (one-tailed test; alpha 5%)
Is there evidence of causal links between age at menarche and other domains of mental health?	H4.1–4a) We hypothesised that age at menarche would show a causal relationship with symptoms in other domains: anxiety (H4.1a), CD (H4.2a), ODD (H4.3a), and ADHD (H4.4a) at age 14	The simulations for this power analysis were essentially identical to H3a (but with different equivalence bounds and the use of two-tailed tests). Results indicated 95% power to detect an average causal effect (using 2SLS) of Cohen’s *D* ≥ 0.2 when the *R*^2^ of the instrument is 0.10 at either simulated sample size, and 80% power to detect Cohen’s *D* ≥ 0.2 with the *R*^2^ of the instrument at ≥ 0.075 for *N* = 9500, as well as with the *R*^2^ of the instrument at ≥ 0.05 for *N* = 10,500	2SLS regression model with covariates and genetically predicted age at menarche included as independent variables and either symptoms of anxiety, CD, ODD, or ADHD at age 14 as the dependent variable Run the same models with other symptom domains at age 8 as dependent variables, as negative control outcomes Perform a test of equivalence for the estimate against *D* = − 0.20–0.20, the equivalence bounds based on our smallest effect size of interest (SESOI) Two-sample MR sensitivity analyses: MR-Egger, MR-PRESSO, weighted median, and contamination mixture	Any significant links between age at menarche and symptoms of anxiety, CD, ODD, or ADHD at age 14 would suggest that causal effects of age at menarche extended to those other symptom domains. Non-significant estimates would suggest an absence of evidence for this If the equivalence test reveals that our estimate is consistent with effects outside of the region of practical equivalence, this would suggest that the association is of a meaningful magnitude Each of the hypotheses 4.1–4a would be supported if (1) the coefficient for the effect of age at menarche on a domain of 14-year symptoms was different from 0 (two-tailed tests, 5% alpha), (2) we failed to reject the null hypothesis that the causal effect in the population was at least as extreme as the SESOI in either direction (two one-tailed tests, 5% alpha), and (3) we failed to reject the null hypothesis that this causal effect in the population was at least as large as the upper bound of the negative control estimate (one-tailed test; alpha 5%) If a hypothesis was supported, consistent results across the two-sample MR sensitivity analyses would provide further evidence of causality
Is there evidence of causal links between age at menarche and other domains of mental health?	H4.1–3b) We hypothesised that age at menarche would show a causal relationship with rates of diagnoses in other domains: anxiety disorders (H.4.1b), conduct disorders (H4.2b) including CD and ODD, and ADHD (H4.3b) during adolescence	The simulations for this power analysis were essentially identical to H3b (but with different equivalence bounds and the use of two-tailed tests). Results indicated 95% power to detect a causal effect (using logistic 2SLS) of Cohen’s *D* ≥ 0.26 when the *R*^2^ of the instrument is ≥ 0.075 and diagnosis prevalence is 14% in either simulated sample size (*N* = 9500 and *N* = 10,500), and 80% power for Cohen’s *D* ≥ 0.20 in the same scenarios	Multivariate logistic (two-stage) regression model with covariates included as independent variables and either diagnoses of anxiety, conduct disorders, or ADHD during adolescence (ages 10–17) as the dependent variable Perform a test of equivalence for the estimate against *D* = − 0.20–0.20, the equivalence bounds based on our smallest effect size of interest (SESOI)	Any significant links between age at menarche and anxiety disorders, disruptive behaviour disorders, or ADHD during adolescence would suggest that causal relationships with age at menarche extended to those other mental health domains. Non-significant estimates would suggest an absence of evidence for this If the equivalence test reveals that our estimate is consistent with effects outside of the region of practical equivalence, this would suggest that the association is of a meaningful magnitude Each of hypotheses 4.1–3b would be supported if (1) the coefficient for the effect of age at menarche on that diagnosis was different from 0 (two-tailed tests, 5% alpha) and (2) we failed to reject the null hypothesis that the causal effect in the population was at least as extreme as the SESOI in either direction (two one-tailed tests, 5% alpha)

#### Software and analysis code

We conducted all statistical analyses in R version 4.1.2. Data preparation and analysis code is publicly available via GitHub: https://github.com/psychgen/aam-psych-adolesc-rr.

#### Overview of hypothesis tests and inference criteria

Pre-specified statistical tests and inference criteria for each hypothesis are summarised in Table 2, including the interpretation of all potential patterns of results. Further details about the main and sensitivity analyses, equivalence bounds, and inference criteria are in Additional file 6. Note that for directional hypotheses, we pre-specified one-sided null hypothesis significance tests and equivalence tests; therefore, some reported *p*-values are one-tailed.

## Results

The pre-registered analyses were conducted according to the stage 1 protocol [91], and all deviations are detailed and justified in Table 3.

**Table 3:** Deviations from the registered stage 1 protocol, following the Preregistration Deviation Table Template (https://osf.io/et6km/)

Deviations						
No	**Details**		**Original wording**	**Deviation description**	**Reader impact**	
1	Type	Variables	In the analysis plan, we stated that we would run negative control MR analyses using diagnoses during childhood as the outcome (ages 0–8). We also proposed to include diagnostic status prior to puberty as a covariate in observational analyses if rates were sufficiently high	Due to the low numbers of cases in childhood (7 depression, 85 anxiety, 21 DBD, and 87 ADHD cases), negative control analyses could not be conducted with these as outcomes. In addition, depression case status prior to puberty could not be included as a covariate	We can assure readers that we proposed to “co-vary for depression status prior to puberty (ages 0–8) if rates of pre-puberty diagnoses are sufficiently high and evenly distributed across levels of the outcome variable to allow model convergence”. This criterion was not met for childhood depression cases	
	Reason	Plan not possible				
	Timing	After data access				
2	Type	Covariates	In the “Methods” section, on the MR models: “We will include covariates in these analyses to increase statistical efficiency, and to control for any residual population stratification”	After seeking expert guidance, we did not include covariates in the MR models, since this may bias results. Including covariates in MR is not common, which was the result of a miscommunication	This deviation avoids introducing bias into the MR analyses (which assume there are no confounders), and so readers should trust results more because of this change	
	Reason	Miscommunication				
	Timing	After results known				
3	Type	Covariates	In the “Methods” section, we stated that we would include the number of children in the household (age 8) as a covariate	Due to a high number of missing values at age 8, parity (based on birth registry data with no values missing) was used instead	This change to a similar but more complete covariate is unlikely to influence results, beyond improving the imputation model performance	
	Reason	Typo/error				
	Timing	After data access				
4	Type	Variables	In the “Methods” section, on two-sample MR instruments: “We will infer the forward strand alleles using allele frequency information for palindromic SNPs (SNPs with minor allele frequency > 0.3 will be discarded, as these cannot be reliably inferred)”	Since the summary statistics did not include the effective allele frequency, we could not infer the forward strand for palindromic SNPs. Instead, we contacted the senior author of the GWAS, who could confirm that the sumstats were formatted to be on the forward strand	Assuming that summary statistics are formatted to be on the forward strand has become a common approach in the field. Moreover, since we knew the age at menarche summary statistics were on the forward strand, this deviation should have little impact on the readers’ interpretation of the results	
	Reason	Plan not possible				
	Timing	After data access				
5	Type	Analysis	We stated that we would run the observational analyses in the largest available sample of 14-year questionnaire responders and restrict these analyses to only genotyped individuals as a sensitivity analysis	We did not restrict to genotyped individuals only, as all variables in the analytic dataset (including the genetic instrument) were multiply imputed. This was according to the stage 1 protocol	Restricting to genotyped individuals only would likely not have been that informative, so this should not affect readers’ interpretation of the results	
	Reason	Typo/error				
	Timing	After data access				
6	Type	Data preparation	We proposed to impute: “early-life diagnoses for the oldest MoBa participants (because linkage is only available since 2008). In addition, at the time of carrying out the analyses, the linked registries will have missing data about diagnoses in the later years of adolescence for younger MoBa participants (because they will not have yet turned 18 by 2021)”	Attempts to multiply impute diagnostic outcomes were unsuccessful, likely due to the sparse and binary nature of the data. Censoring of early-life diagnoses was a minor issue, since numbers were too low for inclusion in analyses. However, the censoring of diagnoses in the later years remains a limitation	Since we did not impute diagnoses, the precision of the MR analyses was lower than anticipated for these outcomes. Readers should take this into account when interpreting the results. Censoring may lead to reduced precision and, potentially, underestimation of effect sizes. This is mentioned as a limitation	
	Reason	Plan not possible				
	Timing	After data access				
7	Type	Analysis	In the protocol, we stated: “When outcomes are excessively skewed (based on the skewness test implemented in the moments package in R [76]) or for binary outcomes (for which a logistic model will be used in the second stage), we will apply a post-estimation correction of the standard errors (the HC1 option in the sandwich R package [77])”	After seeking expert input, we calculated robust standard errors for both continuous and binary outcomes. In addition, non-normality was handled by transforming all symptom outcomes, using log or square root transformation, depending on the impact of each transformation on skewness and kurtosis	These deviations from the registered protocol led to small differences in the results, except for conduct disorder. For this highly skewed outcome, the MR point estimate became more extreme after transformation. Our deviations made sure 2SLS analyses accounted for uncertainty in the first stage and that the data was appropriate for linear regression	
	Reason	New knowledge				
	Timing	After results known				
8	Type	Data preparation	In the section on outliers: “For other phenotype data, values > 4 standard deviations from the mean were treated as outliers and coded as missing (e.g., to remove implausible height/weight values used to calculate BMI)”	According to the quality control procedures for MoBa phenotypic data implemented in the *phenotools* package, we used > 3 standard deviations from the mean to define outliers	This deviation, using a stricter criterion for defining outliers in the calculation of BMI, was very minor and should not influence readers’ interpretation of the results	
	Reason	Others (please explain)				
	Timing	After data access				
9	Type	Variables	We stated: “We will therefore conduct an MVMR analysis with genetic instruments for age at menarche, childhood body size and adult BMI included in the same model”	Since conditional *F*-statistics were less than 10 when including either childhood body size or adult BMI in MVMR analyses, we did not include both as additional instruments due to low power	It should be apparent to the readers that the decision to not add further complexity when simpler models did not satisfy the criterion for instrument strength (*F* > 10) was well justified, to avoid weak instrument bias	
	Reason	Plan not possible				
	Timing	After results known				
10	Type	Analysis	In the analysis plan, we stated that MVMR analyses including BMI and estradiol as additional exposures would be conducted for all other symptom domains, as part of the sensitivity analyses	We did not conduct MVMR analyses with BMI and estradiol as additional exposures for the other symptom domains, since (a) precision was low, (b) one-sample MR estimates were consistent with the null for most domains, and (c) these analyses were especially relevant to depression	This deviation should have a limited impact on readers’ interpretation of the results, since the addition of these analyses would not be very informative, both due to low precision and because the primary interest was in whether BMI confounded the observed causal relationship with depression	
	Reason	Typo/error				
	Timing	After results known				
	Type	Variables	About MVMR: “This will be based on the latest GWAS meta-analysis of major depressive disorder, which identified 223 variants independently associated with depression [72]”	The summary statistics from the GWAS we intended to use were not available; therefore, we used summary statistics from Howard et al. [70] for depression	These summary statistics are similar to what we intended to use, as part of a relatively minor sensitivity analysis and so should not affect readers’ interpretation of the results	
11	Reason	Plan not possible				
	Timing	After data access				

### Descriptive statistics

The average age at menarche after multiple imputation (since 7.25% had not reached menarche at age 14) was 12.69 years (SD = 1.18). We conducted a sensitivity analysis setting an equal number of age at menarche values to missing, testing the imputation accuracy (see Additional file 8). The genetic instrument for age at menarche (based on 235 independent SNPs present in MoBa) explained 6.9% of the variation in age at menarche (*R*^2^ = 0.069, *F* = 996.4) and was associated with BMI at 8 (*β* =  − 0.08, 95% CI [− 0.10, − 0.05], *p* < 0.01) and 14 years (*β* =  − 0.10, 95% CI [− 0.12, − 0.08], *p* < 0.01). There were no notable associations with other covariates (Additional file 9: Table S3). The mean number of depressive symptoms at age 14 was 9.20 (SD = 6.56).

### Depressive symptoms and diagnoses

#### Main analyses of symptom outcomes

**Fig. 3:**
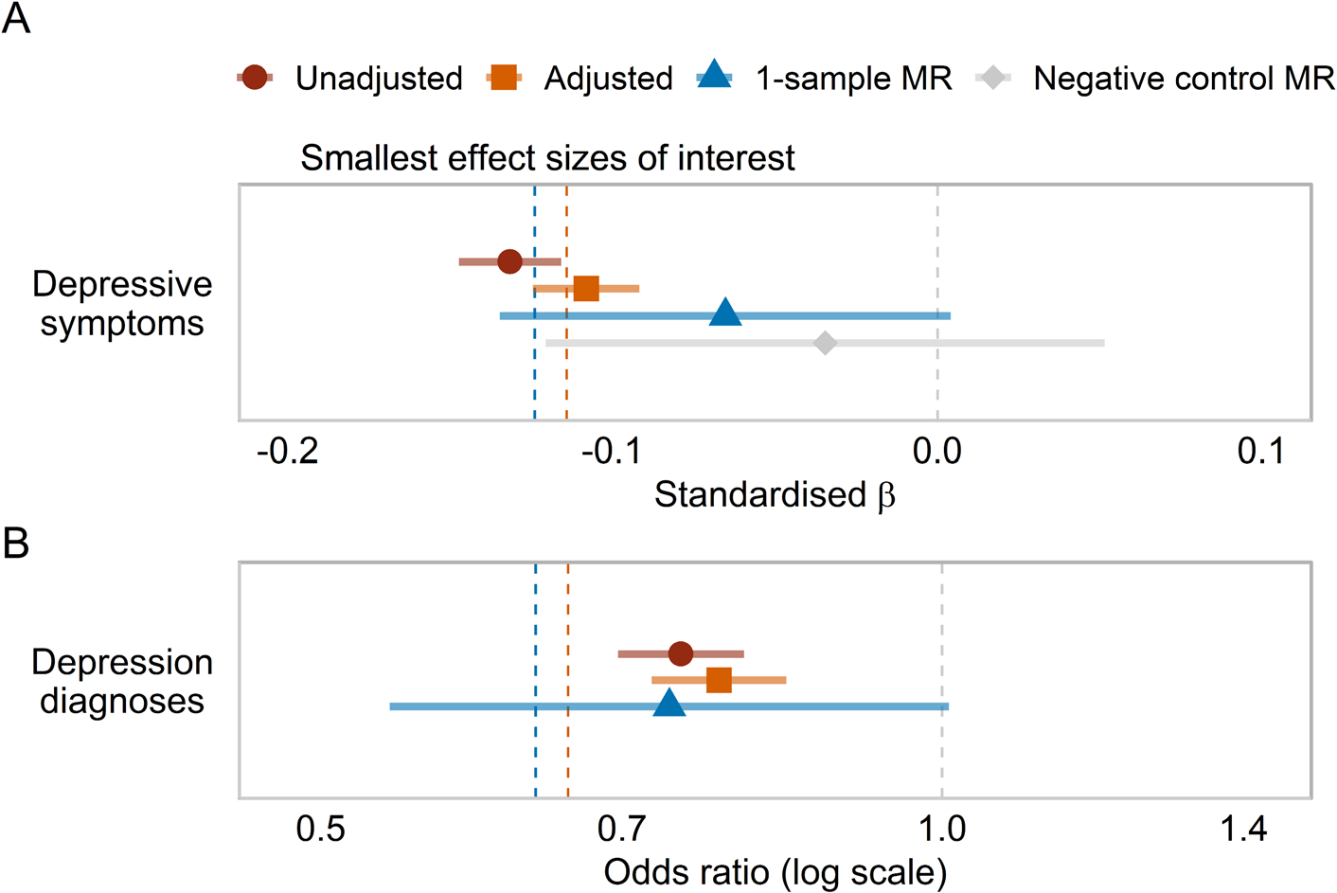
Observational and causal links between age at menarche and depression. **A** Standardised betas of age at menarche predicting adolescent depressive symptoms, based on linear regressions unadjusted and adjusted for covariates and symptoms at age 8, one-sample Mendelian randomisation (MR), and negative control MR with 8-year depressive symptoms as the outcome. **B** Standardised odds ratios of age at menarche predicting depression diagnoses during adolescence, based on logistic regressions unadjusted and adjusted for covariates, and one-sample MR. In both panels, the orange dashed line represents the smallest effect size of interest for the observational analysis; the blue dashed line represents the smallest effect size of interest for the MR. NB: 95% confidence intervals are presented to show the precision of the estimates, but all statistical tests for depression outcomes were pre-specified to be one-tailed, meaning that the visual interpretation of the CIs in relation to the point null and smallest effect sizes of interest differs from the test result (described in text) in places

An earlier age at menarche was observationally associated with more depressive symptoms at age 14 (*β* = ** − **0.11, 95% CI [**− **0.12, − 0.09], *p*_one-tailed_ < 0.01) after adjusting for covariates and pre-pubertal symptoms (see Fig. 3). The one-sample MR analysis indicated a small, causal relationship (*β* = ** − **0.07, 95% CI [**− **0.13, 0.00], *p*_one-tailed_ = 0.03), corresponding to an increase of 0.06 standard deviations in depression symptoms score per earlier year of menarche (after re-scaling the estimate). The adjusted observational estimate was consistent with an effect as extreme as our smallest effect size of interest, but the causal estimate was just within the region of practical equivalence to 0. In our negative control one-sample MR analyses, pubertal timing was not associated with depressive symptoms prior to puberty (*β* = ** − **0.03, 95% CI [**− **0.12, 0.05], *p*_one-tailed_ = 0.22), and the 14-year estimate was consistent with an effect more extreme than this.

#### Main analyses of diagnostic outcomes

For depression diagnoses during adolescence (see Fig. 3B), we also found evidence of a small association in the observational analysis (OR = 0.78, 95% CI [0.72, 0.84], *p*_one-tailed_ < 0.01) and one-sample MR (OR = 0.74, 95% CI [0.54, 1.01], *p*_one-tailed_ = 0.03). The MR estimate was equivalent to a 29% increase in the likelihood of receiving a depression diagnosis with each year of earlier menarche. The number of depression cases in childhood (i.e., 7) was too small to include this as a negative control outcome. Only the MR estimate, which was less precise than the observational estimates, was consistent with values at least as extreme as our SESOI.

#### Sensitivity analyses

**Fig. 4:**
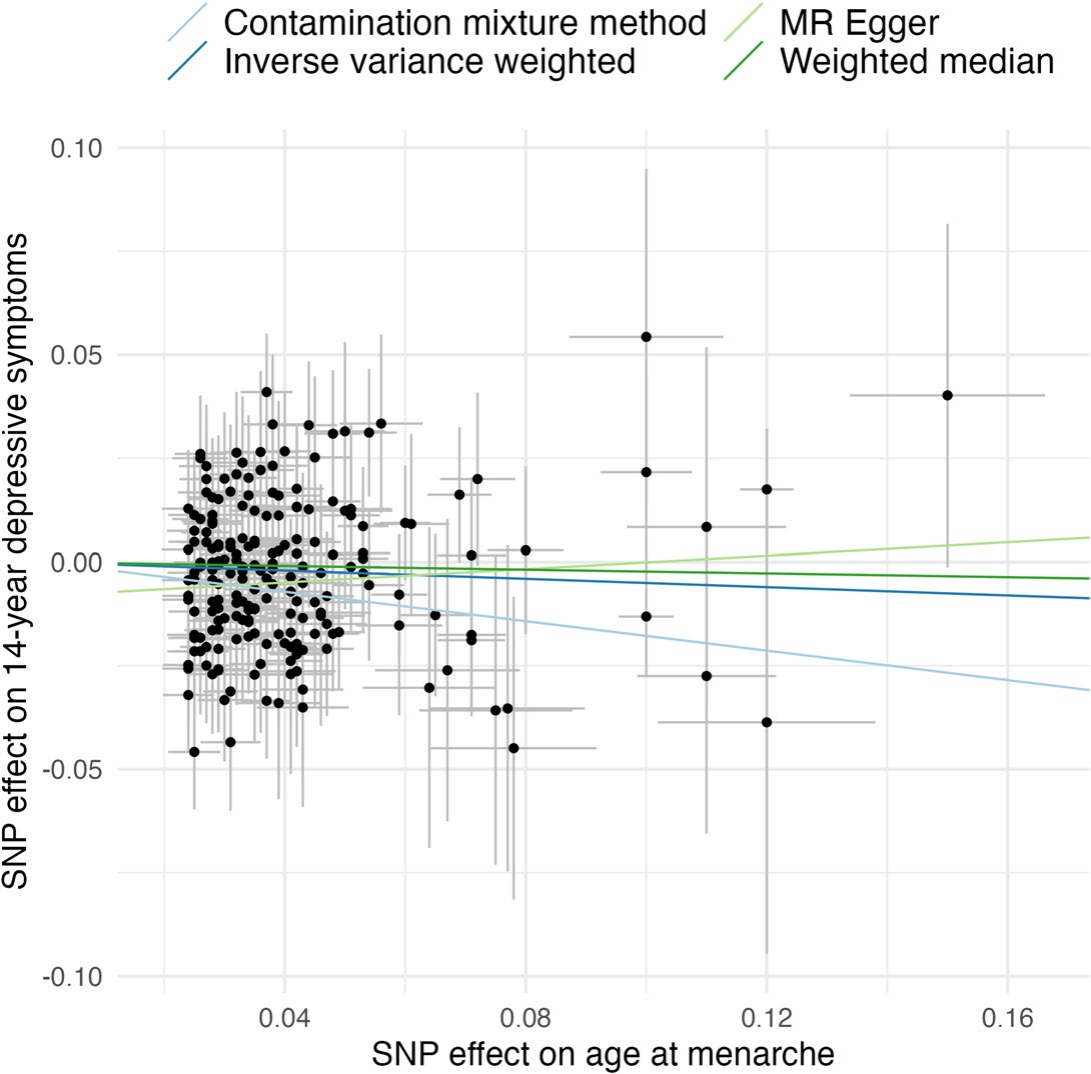
Two-sample MR sensitivity analyses of age at menarche and depressive symptoms. MR sensitivity analyses showing broadly consistent directions of effect (except for MR-Egger), with an earlier age at menarche related to elevated adolescent depressive symptoms. MR, Mendelian randomisation; SNP, single nucleotide polymorphism

The two-sample MR sensitivity analyses with depressive symptoms as the outcome yielded mixed results (see Fig. 4). The IVW (*β* = ** − **0.05, 95% CI [**− **0.11, 0.01], *p*_one-tailed_ = 0.04), contamination mixture (*β* = ** − **0.18, 95% CI [**− **0.28, − 0.04], *p*_one-tailed_ < 0.01), and weighted median methods (*β* = ** − **0.02, 95% CI [**− **0.11, 0.06], *p*_one-tailed_ = 0.31) gave estimates in a consistent direction with the one-sample MR. The MR-Egger estimate was positive (*β* = 0.08, 90% CI [**− **0.07, 0.23], *p*_one-tailed_ = 0.14). Moreover, after Steiger filtering, the IVW estimate was attenuated (*β* = ** − **0.02, 95% CI [**− **0.08, 0.04], *p*_one-tailed_ = 0.22), whereas the contamination mixture estimate was unchanged (*β* = ** − **0.17, 95% CI [**− **0.25, − 0.04], *p*_one-tailed_ = 0.01). The MR-Egger intercept provided little evidence of directional pleiotropy, with an intercept value of − 0.006 (95% CI [**− **0.01, 0.00], *p* = 0.05). The MR PRESSO global test did not detect any outliers.

MVMR analyses accounting for overlap with childhood body size and adult BMI were limited by weak instruments (conditional *F*-statistic range 7.2–10.3). The estimate for age at menarche predicting depressive symptoms at age 14 was in a consistent direction with the IVW estimate (which was *β* =  − 0.05) but partly attenuated when accounting for childhood body size (*β* =  − 0.03, 95% CI [− 0.07, 0.02], *p*_one-tailed_ = 0.11) and substantially attenuated when accounting for adult BMI (*β* =  − 0.01, 95% CI [− 0.05, 0.04], *p*_one-tailed_ = 0.40). Modified *Q*-statistics indicated pleiotropy when including childhood body size (*Q*_1866_ = 2044.3, *p* < 0.01) and adult BMI (*Q*_1812_ = 1924.0, *p* = 0.03). When including genetically predicted estradiol as a second exposure, the estimate was only somewhat attenuated (*β* =  − 0.04, 95% CI [− 0.09, − 0.00], *p*_one-tailed_ = 0.02). Again, there was evidence of pleiotropy (*Q*_1804_ = 1951.0, *p* < 0.01). The MVMR sensitivity analyses provided broadly similar results (Additional file 10: Table S4).

Furthermore, we incorporated the stage of breast development as an additional exposure in the observational models. A more advanced breast stage was associated with more depression diagnoses but including it in the same model left estimates for age at menarche largely unchanged (see Additional file 8). We also conducted the analyses based on a categorised age at menarche exposure and dichotomised SFMQ—as in Sequeira et al. [56]—which showed consistent results (see Additional file 8). Finally, we also incorporated IP weights to account for selective attrition, which attenuated much of the differences in baseline covariates between participants (*n* = 13,398) and non-participants (*n* = 41,832) at age 14 (Additional file 11: Fig. S9). The weighted results for symptoms and diagnoses of depression were similar to the unweighted results, although with somewhat reduced precision due to the inclusion of the weights (Additional file 12: Fig. S10).

### Symptoms and diagnoses in other domains

#### Main analyses of symptom outcomes

**Fig. 5:**
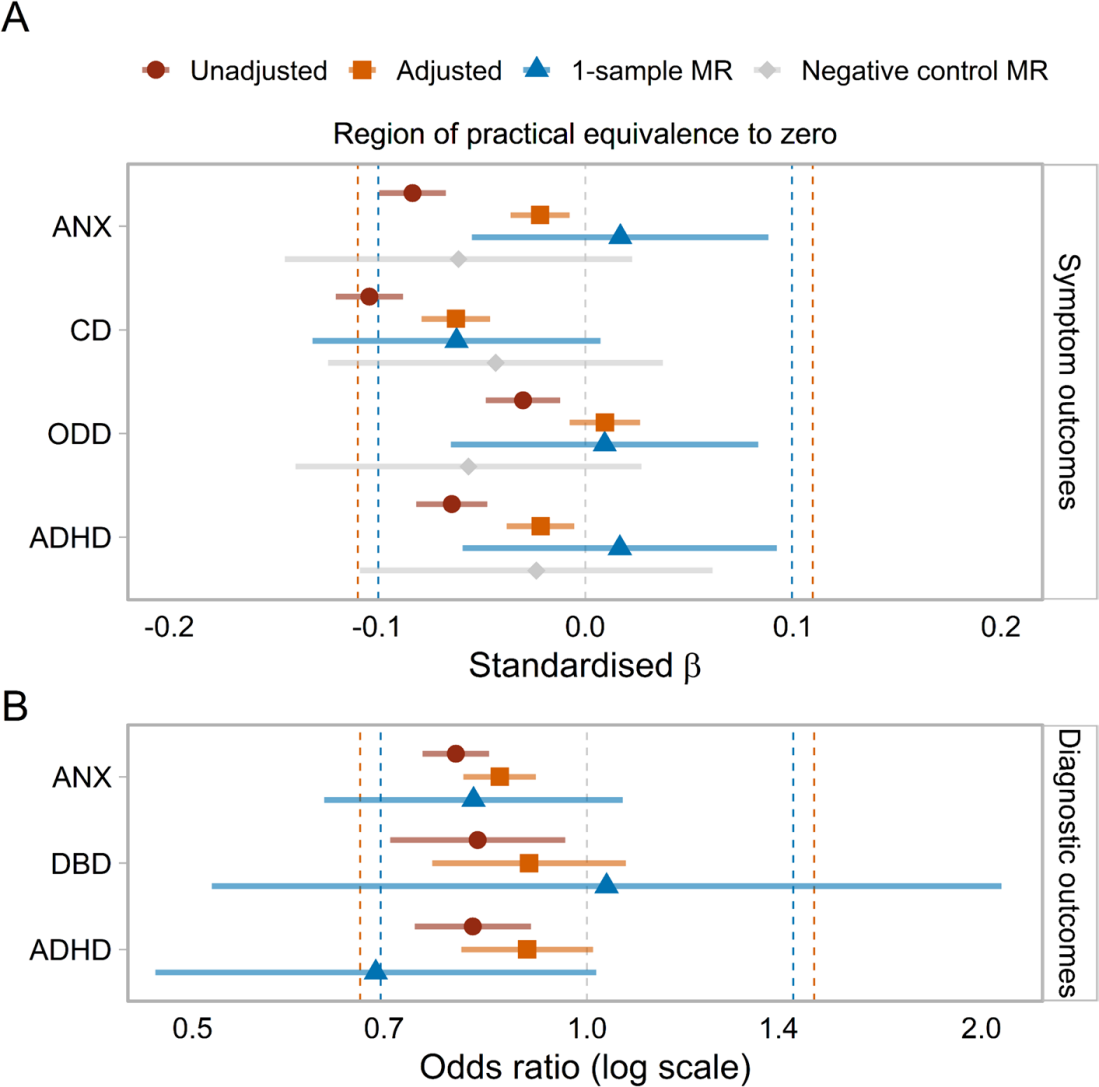
Observational and causal links between age at menarche and other domains. **A** Standardised betas for age at menarche predicting symptoms in other domains of mental health in adolescence, based on linear regressions unadjusted and adjusted for covariates, concurrent depressive symptoms (age 14) and pre-pubertal symptoms (age 8), one-sample Mendelian randomisation (MR), and negative control MR with 8-year symptoms as outcomes. **B** Standardised odds ratios of age at menarche predicting diagnoses in other domains of mental health during adolescence (ages 10–17), based on logistic regressions unadjusted and adjusted for covariates, adolescent (ages 10–17) depression diagnoses and childhood diagnoses for each domain (ages 0–8), and one-sample MR. In both panels, the orange dashed line represents the smallest effect size of interest for the observational analysis; the blue dashed line represents the smallest effect size of interest for the MR; 95% confidence intervals are presented. ANX, anxiety; CD, conduct disorder; ODD, oppositional defiant disorder; ADHD, attention-deficit hyperactivity disorder; DBD, disruptive behaviour disorder

For anxiety symptoms at age 14 (see Fig. 5), the observational analysis adjusted for covariates, concurrent depressive symptoms and pre-pubertal anxiety symptoms showed little evidence of an association (*β* =  − 0.02, 95% CI [− 0.04, − 0.01], *p* < 0.01). Also, the one-sample MR provided no evidence of a causal relationship (*β* = 0.02, 95% CI [− 0.05, 0.09], *p* = 0.64). Similarly, there was little evidence for a relationship with ADHD traits in the fully adjusted observational analysis (*β* =  − 0.02, 95% CI [− 0.04, − 0.01], *p* = 0.01) or the MR (*β* = 0.02, 95% CI [− 0.06, 0.09], *p* = 0.67). There was a small adjusted observational relationship with CD symptoms (*β* =  − 0.06, 95% CI [− 0.08, − 0.05], *p* < 0.01), with which the MR estimate was consistent, but confidence intervals included 0 (*β* =  − 0.06, 95% CI [− 0.13, 0.01], *p* = 0.08). The results for ODD symptoms showed no evidence of an observational (*β* = 0.01, 95% CI [− 0.01, 0.03], *p* = 0.27) or causal relationship (*β* = 0.01, 95% CI [− 0.06, 0.08], *p* = 0.80). Only the unadjusted (but not adjusted) observational and causal estimates for CD symptoms were consistent with effects outside the range of practical equivalence to 0. Negative control analyses for these outcomes were consistent with these results.

#### Main analyses of diagnostic outcomes

For anxiety diagnoses during adolescence (see Fig. 5), the observational analysis adjusting for covariates, adolescent depression, and childhood anxiety diagnoses showed a small observational association (OR = 0.86, 95% CI [0.80, 0.91], *p* < 0.01), and the MR estimate was consistent, but confidence intervals included the null (OR = 0.82, 95% CI [0.63, 1.07], *p* = 0.14). Similarly, age at menarche showed no evidence of an observational (OR = 0.90, 95% CI [0.80, 1.01], *p* = 0.07) or causal relationship with ADHD diagnoses (OR = 0.69, 95% CI [0.47, 1.02], *p* = 0.06), although the MR point estimate was more extreme than the effect found for depression diagnoses. For DBD diagnoses, there was no evidence of an observational (OR = 0.90, 95% CI [0.76, 1.07], *p* = 0.24) or causal relationship (OR = 1.04, 95% CI [0.52, 2.07], *p* = 0.92). Across the observational analyses, all estimates fell within our defined range of practical equivalence to 0, while MR findings were generally too imprecise to draw clear conclusions. There were too few diagnoses in childhood to conduct negative control analyses (see Table 3).

#### Sensitivity analyses

The two-sample MR sensitivity analyses for symptoms in other domains showed little evidence of any associations apart from CD, where all estimates were consistent with earlier age at menarche leading to more CD symptoms (Additional file 13: Fig. S11). MVMR analyses accounting for overlap with major depression provided little evidence of causal relationships between age at menarche and 14-year symptoms of anxiety (*β* =  − 0.01, 95% CI [− 0.05, 0.03], *p* = 0.63), CD (*β* = 0.00, 95% CI [− 0.04, 0.04], *p* = 0.94), ODD (*β* = 0.00, 95% CI [− 0.04, 0.04], *p* = 0.87), and ADHD (*β* = 0.01, 95% CI [− 0.03, 0.05], *p* = 0.80). MVMR sensitivity analyses were sometimes imprecise but consistent with the pattern of null findings (Additional file 10: Table S4). The MR-Egger test showed little evidence of directional pleiotropy for any of the outcomes. The MR PRESSO global test also did not detect any outliers.

### Exploratory analyses

We conducted unregistered follow-up analyses of the causal effect of age at menarche on diagnostic outcomes since the MR results were imprecise. To explore whether the estimates for anxiety and ADHD were attenuated when accounting for comorbid depression and corresponding childhood diagnoses, we ran one-sample MR models with these included as covariates. This somewhat attenuated the estimate for anxiety (OR = 0.86, 95% CI [0.66, 1.13]) but not for ADHD (OR = 0.64, 95% CI [0.43, 0.97]). We also sought to explore the impact of the timing of diagnosis further, since this may have an impact on estimates. To this end, we divided the outcomes into new diagnoses in (1) preadolescence (ages 9–11), (2) early adolescence (ages 12–14), and (3) mid-late adolescence (ages 15–17). For depression, there was only evidence of a causal relationship in early (OR = 0.50, 95% CI [0.26, 0.95]) and not mid-late adolescence (OR = 0.95, 95% CI [0.64, 1.41]). The pattern for ADHD diagnoses was the same, with only a relationship in early adolescence (OR = 0.47, 95% CI [0.23, 0.95]). There were no relationships with anxiety diagnoses in either time window (see Additional file 8).

## Discussion

In this Registered Report, we assessed the causal link between age at menarche and adolescent mental health in a large, population-based cohort. In observational analyses, we found evidence of an association between earlier age at menarche and elevated depressive symptoms at age 14, which was robust to the inclusion of measured covariates, pre-pubertal symptoms, and across all sensitivity analyses. In contrast, age at menarche was not associated with symptoms in the other domains apart from CD, once depressive symptoms were accounted for. One-sample MR analyses mirrored the observational results—with evidence of a small, causal effect of earlier age at menarche on elevated depressive symptoms—but not the other outcome domains. Negative control MR analyses using pre-pubertal symptoms as outcomes corroborated this pattern of findings. The results for diagnostic outcomes were generally less precise, indicating a causal effect of earlier age at menarche on more depression diagnoses during adolescence, but not diagnoses in other domains. Taken together, the main analyses supported our hypothesis of a causal effect of earlier age at menarche on diagnoses of adolescent depression and suggest that this effect is specific to depression, rather than influencing adolescent mental health in general.

Our adjusted observational estimates, negative control analyses, and one-sample MR analyses were all consistent with small causal effects of age at menarche on symptoms and diagnoses of depression. This finding effectively replicates a previous finding in adolescents [56] and aligns with previous findings in adults [61, 70, 92]. For each year earlier menarche, the odds of being diagnosed with depression during adolescence (ages 10–17) increased by approximately 29%. The robustness of this effect—which received broadly consistent empirical support across different outcomes and methodologies—is striking. Nonetheless, some considerations are important to its interpretation. The effects of age at menarche on symptoms and diagnoses of depression were very small, and the extent to which they should be considered clinically meaningful is a matter of debate. On the symptom outcome, individuals with a year earlier menarche would score less than half a point higher, on average, on the SMFQ scale. For diagnoses, the absolute risk of adolescent depression diagnoses changes from 5.2 to 6.6% with 1 year earlier menarche. Our analyses defined a region of practical equivalence to 0 based on pre-defined smallest effect sizes of interest, and while some estimates were consistent with effects outside this region, the majority of plausible values fell within. It should be noted that our smallest effect sizes of interest were set based on existing empirical evidence, and not based on clinical change thresholds—which may be preferable [86]. Overall, the effect sizes obtained here are consistent with the small associations obtained for a range of mental health and behavioural outcomes in other studies [20, 36] and demonstrate a reduction in effect sizes when accounting for confounding.

While the evidence from our main analyses of depression outcomes was relatively consistent, potential complexities in the interpretation of these relationships prompted us to perform extensive sensitivity analyses. The two-sample MR sensitivity analyses, which make different assumptions about the role of horizontal pleiotropy, provided mixed results regarding the causal relationship of age at menarche with depressive symptoms. The results based on Steiger filtering suggested that reverse causation, or other pleiotropic pathways, may be involved in the relationship between age at menarche and adolescent depressive symptoms. It should be noted that these analyses were relatively imprecise because they required that we perform GWAS of the symptom outcomes in MoBa with a sample size (9832) well below what is typically required for genomic discovery. We also conducted multivariable MR analyses, which were designed to estimate the direct effect of age at menarche while accounting for BMI. The results indicated that the association between age at menarche and depressive symptoms was confounded by BMI. The estimate was in a consistent direction but partly attenuated when including childhood body size and markedly attenuated when including adult BMI. While childhood body size is a likely confounder, post-pubertal BMI could also be on the causal pathway to depression; therefore, the impact of adjusting for adult BMI should not be taken primarily as evidence of confounding. These analyses were limited by weak instruments, so may have been biassed towards the null by the violation of the relevance assumption. Nonetheless, these and previous findings [56, 61] are in line with the role of BMI as a confounder of the link between pubertal development and adolescent depression. Future research utilising stronger genetic instruments would be required to quantify the extent of confounding by BMI, and its potential mediating role.

The pattern of results in our study suggests that age at menarche may contribute to symptom differentiation in adolescence, affecting risk for depression diagnoses specifically, rather than acting across the range of mental health outcomes included here. There were some possible exceptions, including conduct symptoms (where the adjusted observational estimate suggested a small effect, and the one-sample MR result was consistent in magnitude and direction but non-significant) and ADHD (where observational results suggested no association, but the MR point estimate—while non-significant—was more extreme than that for depression). However, we saw very little signal for anxiety, disruptive behaviour disorders, and ADHD when accounting for co-occurring depression and other confounders. Previous observational studies have shown associations with a wide range of conditions, including anxiety and behavioural conditions [20, 21]. Notably, our unadjusted estimates for each condition—both symptoms and diagnoses—were also indicative of effects. As a result of the general attenuation of these effects after control for confounders (both explicitly in the adjusted observational estimate and implicitly in MR), we can infer that some of the previously identified associations may not be causal or that they may not remain after accounting for the effect of co-occurring depression. We recommend that future studies account for the comorbidity between mental health conditions when assessing pubertal timing effects, given the potential for condition-specific mechanisms.

Interestingly, sex differences in depression emerge and then peak during adolescence, before declining across adulthood [93]. To explain this phenomenon, future research could benefit from taking a lifespan approach to reproductive development and women’s mental health. Given some evidence of converging aetiology of depression, age at menarche and menopause [70, 94], future studies could aim to explore their joint genetic architecture and potential shared biological underpinnings [95]. A hypothesis that remains to be tested is that the associations between depression and earlier female reproductive events (including age at menarche) may be caused by the duration of sex hormone exposure. If so, we would expect that the risk of depression would increase whenever menarche occurs—and that in the longer term, those with a later menarche would eventually “catch up” with others experiencing it earlier. Indeed, evidence of this “catching up” has recently been found in ALSPAC [96]. In line with this pattern of results, our exploratory analyses indicated an ~ 80% increase in the odds of depression diagnoses and a ~ 90% increase in the odds of ADHD diagnoses per year of earlier menarche in early adolescence (ages 12–14), but not in the years prior to or after this period. This may suggest that menarche causes a transient increase in the prevalence of depression (and possibly ADHD, although this finding should be considered hypothesis-generating rather than conclusive). Future waves of data collection in MoBa could be used to corroborate this further.

We also conducted MVMR analyses utilising genetic variants associated with estradiol as an additional exposure [68], showing limited attenuation of the relationship between earlier age at menarche and elevated depressive symptoms when including estradiol. When including the stage of breast development as an additional predictor in observational analyses, results for depressive symptoms and diagnoses remained unchanged. This conflicts with previous analyses in ALSPAC which suggested that breast stage is driving the association with depressive symptoms rather than age at menarche [42]. To shed some light on this complex pattern of findings, future studies could directly investigate the role of estradiol and other pubertal sex hormones in mediating the effects of pubertal development, rather than relying on proxy measures.

Future studies could also test the causal relationship between pubertal timing and mental health using other genetically informed methods. For instance, co-twin control studies have found that pubertal timing effects on adolescent mental health were largely due to shared genetic influences [97, 98]. MR can also be conducted within families, accounting for population phenomena that may bias genetic associations, such as population stratification, dynastic effects, and assortative mating [99]. Although our sample of female adolescents was too small for conducting well-powered within-family MR, consortium-based analyses could offer a solution.

### Limitations

This study features a relatively large sample of genotyped adolescents and a strong genetic instrument for age at menarche. Furthermore, the Registered Report format and extensive sensitivity analyses strengthen the support for our conclusions. However, there are some important limitations to our study. Although our sample size was larger than previous studies, the precision of MR analyses was low for less prevalent conditions, especially DBD. Another limiting factor to the precision of estimates was the censoring of diagnoses in the registry data, which we proposed to handle with multiple imputation in the stage 1 protocol. However, this turned out not to be feasible, resulting in a deviation from the registered protocol (see Table 3 for a further description and justification). The accuracy of the imputation of missing age at menarche values was also lower than anticipated, but this was a minor issue since values were known to be 15 or higher.

As well as some issues with low precision, limitations in the MR components of our study are linked to the assumptions upon which the method rests. An advantage of our one-sample MR design is that the relevance and independence assumptions could be tested directly. Here, the genetic instrument was strongly associated with age at menarche—but not measured covariates, besides BMI and to some extent maternal age—providing some support for the validity of these assumptions. Furthermore, the two-sample MR sensitivity analyses provided insufficient evidence of directional horizontal pleiotropy, a particularly likely violation of the assumptions in the context of mental health outcomes [100]. However, we reiterate that the final two MR assumptions cannot be verified empirically and that violations may give biassed estimates. It is worth highlighting that the MVMR analyses including BMI may violate these assumptions. First, weak instruments may have biassed the estimates from these analyses towards the null. On a related note, despite broadly consistent results from the MVMR sensitivity analyses, BMI SNPs are highly pleiotropic and may have introduced bias in the MVMR. Overall, the potential of bias means that triangulation is of key importance, and results that are consistent across different methods and outcomes should be given more weight than isolated findings.

Since different methods may lead to different sources of bias, triangulation of multiple analytic approaches has been suggested as a way forward in aetiological epidemiology [58]. However, a key distinction may be between deciding which methods will be combined—and how—before conducting the analyses, or after the fact. “Prospective triangulation”, or pre-specifying a triangulation strategy and specific inference criteria such as in our Registered Report, may further increase the confidence we can have in the results.

There are also limitations to the generalisability of this study. First, MoBa is not fully representative of the general population due to non-random participation at the recruitment stage. Those less represented are the youngest women, those living alone, smokers, and women with previous stillbirths or more than two previous births [101]. However, previous research has suggested that non-random initial participation may have a limited impact on exposure-outcome associations [101, 102]. Furthermore, selective attrition could be expected to have an important impact on our results, due to the substantial drop-out at age 14 in MoBa. Yet, our IPW analyses showed consistent results when accounting for selective attrition. Finally, this study is based on a predominantly white European cohort. Future research in cohorts from non-European countries would advance the field further.

## Conclusions

Our findings—based on extensive analyses and hypotheses registered prior to the availability of data [91]—provided support for the hypothesis that an earlier age at menarche causally increases the risk of adolescent depression. After accounting for depression and other confounders, we found no clear evidence of this effect being present for anxiety, disruptive behaviour disorders, or ADHD. A range of sensitivity analyses corroborated our results but suggested that the causal relationship with depressive symptoms may be partly confounded by BMI and/or influenced by low-level genetic pleiotropy. In sum, although the associations of age at menarche with symptoms and diagnoses of depression are likely partly confounded, our results supported small causal relationships. Since the effects were specific rather than shared across all the mental health domains included here, the timing of menarche may contribute to the developmental differentiation of depression from other mental health conditions in adolescence.

### Supplementary Information

**Additional file 1.** Supplementary methods with information about the: a) categorised age at menarche, b) dichotomised depressive symptoms, c) multiple imputation, d) diagnostic codes, e) inverse probability weighting, f) psychometric properties of scales, g) definition/removal of outliers.

**Additional file 2: Table S1.** With psychometric properties of 8y symptom scales (ordinal Cronbach’s alphas).

**Additional file 3: Table S2.** With overview of variables: details about items, missingness, and processing.

**Additional file 4.** Description of genetic instruments for two-sample MR analyses.

**Additional file 5.** Description of MR sensitivity analyses, including: a) one-sample MR sensitivity analyses, b) two-sample MR sensitivity analyses, c) multivariable MR analyses.

**Additional file 6.** Outline of analyses for each hypothesis, including: a) main analyses, b) negative control analyses, c) the smallest effect size of interest, d) sensitivity analyses, e) inference criteria.

**Additional file 7.** Description of power analyses, including: a) projected prevalence, b) data generation, c) power calculation, d) **Figs. S1-S8** showing results of power analysis for all hypotheses.

**Additional file 8.** Supplementary results, including: a) imputation of age at menarche, b) breast stage as an additional exposure, c) categorised age at menarche, d) exploratory analyses.

**Additional file 9: Table S3.** With associations of genetic instrument for age at menarche with the covariates.

**Additional file 10: Table S4.** With results of multivariable Mendelian randomisation sensitivity analyses.

**Additional file 11: Fig. S9.** With differences between participants and non-participants with IP weighting.

**Additional file 12: Fig. S10.** Showing the impact of inverse probability weighting on results for depression.

**Additional file 13: Fig. S11.** With 2-sample MR sensitivity analyses for other mental health domains.

## Data Availability

The MoBa data are not publicly available as the consent given by the participants does not open for storage of data on an individual level in repositories or journals. Researchers who want access to data sets for replication should submit an application to datatilgang(at)fhi.no. Access to datasets requires approval from the Regional Committee for Medical and Health Research Ethics in Norway and an agreement with MoBa. Data preparation and analysis code for all elements of the project are publicly available on GitHub at https://github.com/psychgen/aam-psych-adolesc-rr.

## Abbreviations

2SLS: Two-stage least squares
ADHD: Attention-deficit hyperactivity disorder
ALSPAC: Avon Longitudinal Study of Parents and Children
BMI: Body mass index
CD: Conduct disorder
CI: Confidence interval
GWAS: Genome-wide association study
IPW: Inverse probability weighting
IVW: Inverse variance weighted
KUHR: Control and payment of health refunds
MI: Multiple imputation
MoBa: Norwegian Mother, Father, and Child Cohort Study
MR: Mendelian randomisation
MR-Egger: Mendelian randomisation-Egger
MR-PRESSO: MR pleiotropy residual sum and outlier
MVMR: Multivariable Mendelian randomisation
NHST: Null hypothesis significance testing
NPR: Norwegian Patient Registry
ODD: Oppositional defiant disorder
RCT: Randomised controlled trial
RS-DBD: Rating Scale for Disruptive Behaviour Disorders
SCARED: Screen for Child Anxiety-Related Disorders
SESOI: Smallest effect size of interest
SMFQ: Short Mood and Feelings Questionnaire
SNPs: Single nucleotide polymorphisms
