# Supplementary material for: Assessing causal links between age at menarche and adolescent mental health: a Mendelian randomisation study
Source: BMC Med. 2024 Apr 12;22:155. doi: 10.1186/s12916-024-03361-8 (PMC11015655; doi:10.1186/s12916-024-03361-8)
Supplement: Supplementary file 1 — Additional file 1. Supplementary methods with information about the: a) categorised age at menarche, b) dichotomised depressive symptoms, c) multiple imputation, d) diagnostic codes, e) inverse probability weighting, f) psychometric properties of scales, g) definition/removal of outliers. [file 12916_2024_3361_MOESM1_ESM.docx]

## **Additional file 1: Supplementary Methods**

### ***Categorised age at menarche***

For the categorised version of the exposure, the grouping into ‘early’ (≤ 1 SD below the mean), ‘average’ (> 1 SD below the mean and < 1 SD above the mean) and ‘late’ onset (≥ 1 SD above the mean) was based on the distribution of age at menarche in our data.

### ***Dichotomised depressive symptoms***

To facilitate replication of Sequeira et al., the SMFQ was also dichotomised (with high depressive symptoms defined as scoring 16 or above, to match the prevalence of ~15.5% cases based on a cut-off of 11 in ALSPAC).

### ***Multiple imputation***

We included all variables in the analytic dataset (limited to 14-year questionnaire returners) in the multiple imputation - imputing the exposures, covariates, and symptom outcomes. Age at menarche values were conditionally imputed with a lower bound set to age 15 for those reporting not yet having had their first period (n = 769), and other missing values imputed freely (n = 203). In addition, to aid the imputation of age at menarche above age 14 we added self-reported growth spurts, body hair development, and skin changes as additional indicators of pubertal stage (using a scale from 0-3, from ‘not yet started’ to ‘already complete’). To validate this approach, we also set an equivalent percentage of randomly selected individuals to missing, imputing their age at menarche and comparing the imputed values to their actual values. We performed multiple imputation using the *mice* package in R, creating 50 datasets. Estimates were then pooled across the datasets using Rubin’s rules.

### ***Diagnostic codes***

We linked to the Control and payment of health refunds (KUHR) and Norwegian Patient Registry (NPR) registries to obtain diagnoses from medical records. KUHR covers primary health care (using codes from The International Classification of Primary Care; ICPC-2), whereas NPR covers all public specialist health-care services in Norway (using codes from ICD-10). We extracted information on diagnoses of depressive disorders (ICPC-2: P76; ICD-10: F32-F33, F34.1), anxiety disorders (ICPC-2: P74, P79, P82; ICD-10: F40-F44, F93.0-F93.2), ADHD (ICPC-2: P81; ICD-10: F90), and conduct disorders (including both CD and ODD; ICPC-2: P23; ICD-10: F91-F92).

### ***Inverse probability weighting***

IP weights were generated based on the results of logistic regression models run in the whole MoBa sample, using return of the 14-year questionnaire participation as the outcome, and all baseline covariates and registry diagnoses (i.e., any variables from the main analytic dataset that do not rely in participation in MoBa beyond the first wave of data collection) as predictors. The models were run in 50 multiply imputed datasets and derived weights subsequently smoothed by averaging across datasets. Predicted probabilities of participation at 14 years were converted to stabilised weights using the following formula:

$$w(x)=\frac{P(participation=1)}{p(x)}$$

### ***Psychometric properties***

Mothers responded to items on the SMFQ and SCARED using a 3-point scale ranging from “Not true” to “True” to describe their children’s symptoms (e.g., depression: “Felt miserable or unhappy”, “Thought s/he could never be as good as other kids”; and anxiety respectively: “My child gets really frightened for no reason at all”). In addition, children responded to the SMFQ items at age 14 using the same scale. The original response set for the RS-DBD items was “Never/rarely”, “Sometimes”, “Often”, and “Very often”, as mothers rated how often their children engaged in different behaviours. An exception was the items for conduct disorder which the adolescents responded to at age 14, on a 6-point scale to obtain more variance. Internal consistency for the SCARED, SMFQ, and RS-DBD subscales at age 8 was generally good to excellent (*N* ⋍ 20,000; see Additional file 2).

### ***Definition/removal of outliers***

We retained all valid responses to the questionnaires. If participants ticked multiple boxes on a single-response item, their response on this item was set to missing. If respondents completed less than half of the items for a given scale, their scale score was not computed and their data for this variable was considered missing. For other phenotype data, values > 3 standard deviations from the mean (this was a deviation, see Table 3) were treated as outliers and coded as missing (e.g., to remove implausible height/weight values used to calculate BMI).
