# Supplementary material for: Assessing causal links between age at menarche and adolescent mental health: a Mendelian randomisation study
Source: BMC Med. 2024 Apr 12;22:155. doi: 10.1186/s12916-024-03361-8 (PMC11015655; doi:10.1186/s12916-024-03361-8)
Supplement: Supplementary file 2 — Additional file 2: Table S1. With psychometric properties of 8y symptom scales (ordinal Cronbach’s alphas). [file 12916_2024_3361_MOESM2_ESM.docx]

## **Additional file 2: Psychometric properties**

**Table S1. Ordinal Cronbach’s alphas for 8-year symptom scales.**

| **Variable** | **Ordinal alpha** |
| --- | --- |
| adhd | 0.94 |
| cd | 0.93 |
| odd | 0.91 |
| dep | 0.92 |
| anx | 0.76 |

adhd, attention-deficit hyperactivity disorder; cd, conduct disorder; odd, oppositional defiant
disorder; dep, depression; anx, anxiety.
