# Supplementary material for: Assessing causal links between age at menarche and adolescent mental health: a Mendelian randomisation study
Source: BMC Med. 2024 Apr 12;22:155. doi: 10.1186/s12916-024-03361-8 (PMC11015655; doi:10.1186/s12916-024-03361-8)
Supplement: Supplementary file 3 — Additional file 3: Table S2. With overview of variables: details about items, missingness, and processing. [file 12916_2024_3361_MOESM3_ESM.docx]

## **Additional file 3: Variable overview**

**Table S2. Overview of variables to be included in the study, with details about items, reporters, and variable processing.**

| **Variable** | **Role** | **Measure** | **Items** | **% missing** | **Processing** |
| --- | --- | --- | --- | --- | --- |
| Age at menarche | Predictor | Single item | 1 | 7.25 | menarche score based on self-reported year of menarche at age 14y |
| Breast stage | Predictor | Single item | 1 | 1.37 | breast stage score calculated based on self-reported values on a scale from 0-4, from ‘not yet started’ to ‘already complete’ at age 14y |
| Growth spurts | Auxiliary | Single item | 1 | 0.99 | growth spurts score calculated based on self-reported values on a scale from 0-4, from ‘not yet started’ to ‘already complete’ at age 14y |
| Body hair development | Auxiliary | Single item | 1 | 1.47 | body hair score calculated based on self-reported values on a scale from 0-4, from ‘not yet started’ to ‘already complete’ at age 14y |
| Skin changes | Auxiliary | Single item | 1 | 1.19 | skin change score calculated based on self-reported values on a scale from 0-4, from ‘not yet started’ to ‘already complete’ at age 14y |
| Body mass index | Covariate | Single items | 2 | 8y: 36.97 14y: 10.40 | bmi scores calculated as weight (kg)/height^2^ (m), based on mother report at age 8y and self-report at age 14y |
| Parental income | Covariate | Single items | 2 | 3.10 | income score calculated as mean of maternal and paternal gross income reported by mother at week 17 of pregnancy |
| Parental education | Covariate | Single items | 2 | 2.97 | education score calculated as mean of maternal and paternal education reported by mother at week 17 of pregnancy |
| Paternal age | Covariate | MBRN | 1 | 0.34 | paternal age at the time of delivery based on registry data |
| Maternal age | Covariate | MBRN | 1 | 0.06 | maternal age at the time of delivery based on registry data |
| Child age | Covariate | Single items | 2 | 8y: 28.03 14y: 0.81 | age at questionnaire completion at age 8y, 14y |
| Financial problems | Covariate | Single item | 1 | 18.17 | financial problems based on mother report at child age 18 months |
| Parental cohabitation | Covariate | Single items | 2 | 18m: 13.35 3y: 23.07 | parental cohabitation based on mother report at child age 18 months, 3y |
| Parity | Covariate | MBRN | 1 | 0 | number of previous deliveries, as registered in MBRN |
| Prenatal depression | Covariate | SCL-5+ SCL-8 | 5 & 8 | w17: 3.00 w30: 4.53 | prenatal score as mean (available items)*total items (IF available_items > total_items/2) reported by mother week 17 & 30 of pregnancy |
| Postnatal depression | Covariate | EPDS | 6 | 7.35 | postnatal score calculated as mean (available items)*total items (IF available_items > total_items/2) reported by mother at 6 months |
| Depressive symptoms | Outcome | SMFQ | 13 | 8y: 27.44 14y: 0 | depression score calculated as mean (available items)*total items (IF available_items > total_items/2) at 8y (mother) & 14y (self-report) |
| Anxiety symptoms | Outcome | SCARED | 5 | 8y: 27.34  14y: 0.49 | anxiety scores calculated as mean (available items)*total items (IF available_items > total_items/2) at 8y (mother) & 14y (self-report) |
| ADHD traits | Outcome | RS-DBD | 18 | 8y: 27.37 14y: 20.45 | adhd scores calculated as mean (available items)*total items (IF available_items > total_items/2) reported by mother at age 8y & 14y |
| CD symptoms | Outcome | RS-DBD | 8 | 8y: 27.37 14y: 0.09 | conduct scores calculated as mean (available items)*total items (IF available_items > total_items/2) at 8y (mother) & 14y (self-report) |
| ODD symptoms | Outcome | RS-DBD | 8 | 8y: 27.38  14y: 20.47 | oppositional scores calculated as mean (available items)*total items (IF available_items > total_items/2) reported by mother at 8y & 14y |
| Diagnoses | Outcome | KUHR/NPR |  |  | ICPC-2: P23, P74, P76, P79, P81, P82 / ICD-10: F32-F33, F34.1, F40-F44, F90, F91-F92, F93.0-F93.2 |

BMI, body mass index; MBRN, The Medical Birth Registry; SCL, The (Hopkins) Symptom Checklist; EPDS, Edinburgh Postnatal Depression Scale; SMFQ, Short Mood and Feelings Questionnaire; SCARED, Screen for Child Anxiety and Related Disorders; ADHD, attention-deficit hyperactivity disorder; CD, conduct disorder; ODD, oppositional defiant disorder; RS-DBD, Parent/Teacher Rating Scale for Disruptive Behaviour Disorders; KUHR, Control and payment of health refunds; NPR, Norwegian Patient Registry; ICPC-2, The International
Classification of Primary Care; ICD-10, International Statistical Classification of Diseases and Related Health Problems.
