# Supplementary material for: Assessing causal links between age at menarche and adolescent mental health: a Mendelian randomisation study
Source: BMC Med. 2024 Apr 12;22:155. doi: 10.1186/s12916-024-03361-8 (PMC11015655; doi:10.1186/s12916-024-03361-8)
Supplement: Supplementary file 4 — Additional file 4. Description of genetic instruments for two-sample MR analyses. [file 12916_2024_3361_MOESM4_ESM.docx]

### **Additional file 4: Two-sample MR instruments**

For the two-sample MR, the SNPs were harmonised using *MR-Base* (71), assuming that the summary statistics were formatted to be on the forward strand (option 1), after consulting with the GWAS senior author (this was a deviation from the protocol, see Table 3). For two-sample multivariable MR analyses accounting for estradiol levels, we used a recently published GWAS in UK Biobank (*N* = 163,985 females of European ancestry) which identified 4 SNPs independently associated with estradiol (at *P* < 1 x 10^-7^) (72). We also conducted two-sample MR analyses to account for potential confounding by BMI, for which the most recent female-only GWAS summary data was used. This was due to the important assumption in two-sample MR that the employed samples stem from the same underlying population. Because of sample overlap between MoBa and the most recent GWAS of BMI in children (which identified 15 SNPs associated with childhood BMI (73)), we used the GWAS of recalled body size at age 10 from the UK Biobank (*N* = 246,511 females), which identified 135 SNPs independently associated with comparative early life body size (69). For adult BMI, we also used GWAS summary data from the UK Biobank (*N* = 246,511 females), which identified 215 SNPs associated with adult-measured BMI (69). The use of these measures as indicators of separate exposures has previously been validated in ALSPAC and employed in an MVMR setting (69). Finally, to mirror how co-occurring depression is accounted for in the observational analyses of other mental health domains, we ran MVMR including a genetic instrument for depression. This was based on a GWAS meta-analysis of major depression (*N* = 807,553), which identified 102 variants independently associated with depression (70). This was a deviation since the summary statistics we intended to use were not available; see Table 3.

To enable running the two-sample MR analyses, we obtained summary statistics for the 14-year symptom outcomes in MoBa by running GWAS in *REGENIE* (74). *REGENIE* is a linear mixed model method which fits a whole-genome (linear) regression model for the phenotype and handles relatedness in the sample. Genotyping batch and the first 20 principal components were included as covariates in the GWAS.
