# Supplementary material for: Assessing causal links between age at menarche and adolescent mental health: a Mendelian randomisation study
Source: BMC Med. 2024 Apr 12;22:155. doi: 10.1186/s12916-024-03361-8 (PMC11015655; doi:10.1186/s12916-024-03361-8)
Supplement: Supplementary file 5 — Additional file 5. Description of MR sensitivity analyses, including: a) one-sample MR sensitivity analyses, b) two-sample MR sensitivity analyses, c) multivariable MR analyses. [file 12916_2024_3361_MOESM5_ESM.docx]

## **Additional file 5: MR sensitivity analyses**

### One-sample MR sensitivity analyses

We carried out linear regression analyses between the genetic instrument and the covariates used for the one-sample MR. The measured covariates serve as proxies for some of the potential confounders of the relationship between the exposure and outcome. If the genetic instrument is associated with a confounder, the second assumption of MR is violated. We also carried out linear regression analysis of the exposure on the genetic instrument for age at menarche and calculated the *F*-statistic to evaluate the relevance assumption. A common rule of thumb is that if *F* < 10, the instrument is considered weak. Here, the criterion of *F* > 10 for age at menarche must be met to successfully test the hypotheses that are based on one-sample MR (H3a, H3b, H4.1-4a, and H4.1-3b). In Sequeira et al. this value was *F* = 114.9, indicating a very strong instrument (based on a previous GWAS of age at menarche).

### Two-sample MR sensitivity analyses

We expected pleiotropic effects in our study, which means that the employed genetic variants affect multiple biological pathways. Specifically, pleiotropic effects via BMI are likely. Vertical pleiotropy, where the genetic variants affect other traits through the exposure, is consistent with a causal interpretation (79). On the other hand, horizontal pleiotropic effects could bias our findings, as they would violate the instrumental variable assumptions. Horizontal pleiotropy means that the genetic variants affect the outcome through other pathways than the exposure of interest.

We employed a standard battery of two-sample sensitivity analyses, which have been developed mainly to address the third MR assumption: that the SNPs used to instrument the exposure only influence the outcome through the exposure. The standard IVW method assumes that there is no directional horizontal pleiotropy (where pleiotropic effects are biasing the estimate in the same direction), which is an assumption that is likely to be violated. Mendelian randomisation-Egger (MR-Egger) regression allows all instruments to be subject to horizontal pleiotropy but has the lowest power to detect a causal effect among these approaches (79). The MR-Egger intercept can be used to assess bias from directional horizontal pleiotropy. MR pleiotropy residual sum and outlier (MR-PRESSO) is also used to test for the presence of directional pleiotropy, and additionally detect outliers (80). The MR-PRESSO test consists of three parts: a) the global test, which detects horizontal pleiotropy, b) the outlier-corrected causal estimate, which corrects for any detected pleiotropy, and c) the distortion test, which tests whether the MR estimate is significantly different after adjustment for outliers. In addition, we used the weighted median estimator, which assumes that the genetic variants representing over 50% of the weight in the analysis are valid instruments (81). The contamination mixture method assumes that only some of the genetic variants are valid instruments and has a well-controlled type 1 error rate compared to similar approaches (82). These five methods make different (to some extent orthogonal) assumptions about horisontal pleiotropy.

### Multivariable MR

It is also possible to estimate the effects of multiple exposures simultaneously, using MVMR (83). Including multiple genetic instruments in the same analysis is valuable when the exposures being studied are highly correlated. Here, we used summary data and the *MVMR* package (https://github.com/WSpiller/MVMR/) to estimate the direct causal effect of age at menarche on mental health while adjusting for BMI. Within the MVMR setting, the first MR assumption is that the instrument is robustly associated with the exposure, conditional on the remaining exposures in the model (84). This is quantified by the modified *Q* statistic (estimating heterogeneity in gene-exposure effects), where a higher degree of heterogeneity indicates greater instrument strength. We converted *Q* to the conventional *F* statistic to evaluate conditional instrument strength (with a threshold of *F* > 10 for each instrument). The second and third assumptions in MVMR are direct extensions of the univariate MR assumptions described above. We employed MVMR-Egger, MVMR-Median, and MVMR-Lasso (85) as sensitivity analyses to test for violations of these assumptions in the multivariable setting.
