# Supplementary material for: Assessing causal links between age at menarche and adolescent mental health: a Mendelian randomisation study
Source: BMC Med. 2024 Apr 12;22:155. doi: 10.1186/s12916-024-03361-8 (PMC11015655; doi:10.1186/s12916-024-03361-8)
Supplement: Supplementary file 6 — Additional file 6. Outline of analyses for each hypothesis, including: a) main analyses, b) negative control analyses, c) the smallest effect size of interest, d) sensitivity analyses, e) inference criteria. [file 12916_2024_3361_MOESM6_ESM.docx]

# **Additional file 6: Outline of analyses**

Table of contents

[Question 1: To what extent is age at menarche associated with adolescent depression?](#_Toc159590894)

[Main analyses](#_Toc159590895)

[The smallest effect size of interest](#_Toc159590896)

[Sensitivity analyses](#_Toc159590897)

[Inference criteria](#_Toc159590898)

[Question 2: Does age at menarche associate with symptoms or diagnoses in other domains, independent of depression?](#_Toc159590899)

[Main analyses](#_Toc159590900)

[The smallest effect size of interest](#_Toc159590901)

[Inference criteria](#_Toc159590902)

[Question 3: What is the evidence for a causal link between age at menarche and depression?](#_Toc159590903)

[Main analyses](#_Toc159590904)

[Negative control outcome analyses](#_Toc159590905)

[The smallest effect size of interest](#_Toc159590906)

[Sensitivity analyses](#_Toc159590907)

[Inference criteria](#_Toc159590908)

[Question 4: Is there evidence of causal links between age at menarche and other domains of mental health?](#_Toc159590909)

[Main analyses](#_Toc159590910)

[The smallest effect size of interest](#_Toc159590911)

[Inference criteria](#_Toc159590912)

##

## Question 1: To what extent is age at menarche associated with adolescent depression?

### Main analyses

*H1a.* First, we hypothesised that earlier age at menarche would be associated with elevated depressive symptoms at age 14. We ran a linear regression model with age at menarche as the independent variable and depressive symptoms at age 14 as the dependent variable. We then added 8-year depressive symptoms as a covariate, to examine whether age at menarche was associated with post-pubertal symptoms independent of pre-pubertal symptoms.

*H1b.* We hypothesised that earlier age at menarche would be associated with higher rates of depression diagnoses during adolescence. We ran a logistic regression model with age of menarche as the independent variable and depression diagnosis as the dependent variable. This analysis included any depression diagnosis in either primary or secondary health care during adolescence (age 10-17).

### The smallest effect size of interest

In line with our directional hypothesis and one-tailed null hypothesis significance test, we applied an equivalence test only using a lower bound (sometimes called an “inferiority” test). This tests whether the null hypothesis of an effect at least as large as the smallest effect size of interest (SESOI) can be rejected. We derived the SESOI for this analysis based on the lower end of the confidence interval of a meta-analytic estimate (as recommended by Lakens, Scheel, and Isager (86)) of age at menarche and depressive symptoms in adolescents. This conservative approach was deemed appropriate given the potential for bias in the literature (estimated to have a small and positive impact on the magnitude of results for early pubertal timing and internalising behaviours in the meta-analysis by Ullsperger and Nikolas (20)). Because they did not estimate the specific association with depression, we conducted a meta-analysis of the studies of community adolescents that they included (24,29,31,38,49) which had data on age at menarche and depressive symptoms (the script used to run the meta-analysis can be found here: <https://github.com/psychgen/aam-psych-adolesc-rr>). The pooled association of 5 eligible studies of age at menarche and depressive symptoms in early-to-mid adolescence was *D* = 0.28 (95% CI = 0.23 - 0.33). Our SESOI was the lower CI bound of this estimate (i.e., *D* = 0.23).

### Sensitivity analyses

*Stage of breast development.* We ran a linear regression with the stage of breast development instead of age at menarche included as the predictor, and then a multiple regression with both breast stage and age at menarche included as predictors, replicating previous observational analyses in ALSPAC (42). Both models were adjusted for 8-year depressive symptoms. Breast stage is an indicator of pubertal stage, which at any point in time during adolescence will be more advanced in those who began puberty earlier (i.e., those with earlier age at menarche). Thus, we first investigated whether breast stage was associated with 14-year depressive symptoms in isolation, and then the relative contribution of each in a multiple regression model including both breast stage and age at menarche. This sensitivity analysis was repeated for depression diagnoses in H1b.

*Categorised exposures/outcomes.* We also ran the same analyses with a categorised version of age at menarche as the exposure and a dichotomised version of the SMFQ as the outcome, as in Sequeira et al. (56). In addition, the categorised version of age at menarche was used for depression diagnoses in H1b.

### Inference criteria

*H1a.* Support for hypothesis 1a was assessed based on whether or not 1) the coefficient for the effect of age at menarche on 14-year depressive symptoms was significantly less than zero (one-tailed test; alpha 5%) in the pre-pubertal symptoms-adjusted model; and 2) we failed to reject the null hypothesis that this effect in the population was at least as large as the SESOI (one-tailed test; alpha 5%).

*H1b.* Support for hypothesis 1b was assessed based on whether or not 1) the coefficient for the effect of age at menarche on odds of depression diagnoses was significantly less than zero (one-tailed test; alpha 5%); and 2) we failed to reject the null hypothesis that this effect in the population is at least as large as the SESOI (one-tailed test; alpha 5%).

## Question 2: Does age at menarche associate with symptoms or diagnoses in other domains, independent of depression?

### Main analyses

*H2.1-4a.* We hypothesised that the association with age at menarche would extend to other symptom domains: anxiety (H2.1a); CD (H2.2a); ODD (H2.3a); and ADHD (H2.4a). To test each hypothesis, we ran linear regression models to examine associations between age at menarche and each symptom domain at age 14. We then added depressive symptoms at age 14 as a covariate in each model to examine whether any associations in other domains were independent of co-occurring depressive symptoms. Finally, we also added a measure of each symptom domain at age 8 as a covariate in the age 14 model for that domain, to examine whether associations between age at menarche and post-pubertal symptoms were additionally independent of pre-pubertal symptoms (these are referred to as the “fully adjusted” models below).

*H2.1-3b.* We hypothesised that the association with earlier age at menarche would extend to diagnoses in other mental health domains: anxiety disorders (H2.1b); DBD, including CD and ODD (H2.2b); and ADHD (H2.3b). For these analyses, we ran logistic regression models to examine associations between age at menarche and odds of receiving each diagnosis during adolescence (age 10-17). We first added depression diagnostic status (age 10-17) as a covariate, then pre-pubertal diagnostic status (age 0-8) in each relevant domain to each model, to examine whether associations with age at menarche were independent of both comorbid depression and prior diagnoses.

### The smallest effect size of interest

We used the lower end of the CIs of a meta-analytic estimate of age at menarche and general psychopathology in adolescents to determine the SESOI across domains because precise meta-analytic estimates were not available or feasible to derive for each of the domains (e.g., there were few previous studies of ADHD). The pooled association of 42 studies in Ullsperger and Nikolas (20) was *D* = 0.27 (95% CI = 0.22 - 0.31). Based on the lower end of the CIs we used equivalence bounds of -0.22 - 0.22 in our analysis.

### Inference criteria

*H2.1-4a.* Support for hypotheses 2.1-4a was assessed based on whether or not 1) the coefficient for the association between age at menarche and a domain of 14-year symptoms in the fully adjusted model was different from zero (two-tailed tests, 5% alpha); and 2) we failed to reject the null hypothesis that the association in the population was at least as extreme as the SESOI in either direction (two one-tailed tests, 5% alpha).

*H2.1-3b.* Support for hypotheses 2.1-3b was assessed based on whether or not 1) the coefficient for the association between age at menarche and a diagnosis in the fully adjusted model was different from zero (two-tailed tests, 5% alpha); and 2) we failed to reject the null hypothesis that the association in the population was at least as extreme as the SESOI in either direction (two one-tailed tests, 5% alpha).

## Question 3: What is the evidence for a causal link between age at menarche and depression?

### Main analyses

*H3a.* We hypothesised that earlier age at menarche would show a causal relationship with elevated depressive symptoms at age 14. In the one-sample MR analysis, we used 2SLS regression to test the relationship between the genetic instrument for age at menarche and depressive symptoms at age 14 (further described in Methods). As in H1a, we again tested on an equivalence bound at the SESOI only in the predicted direction.

To test for bias from unmeasured confounding, we conducted a negative control MR analysis using depressive symptoms prior to puberty (at 8 years) as the outcome. A relationship between genetically predicted age at menarche and childhood depressive symptoms would be temporally implausible, indicating unmeasured confounding. To formally test whether the extent of observed confounding was sufficient to account for the observed effect at 14-years, we subjected the 14-year effect to an equivalence test, setting the equivalence bound to the lower bound of the 8-year estimate.

*Hypothesis 3b.* We further hypothesised that earlier age at menarche would result in higher rates of depression diagnoses during adolescence. As for hypothesis 1b, we ran these binary outcome MR analyses with individuals diagnosed either in primary or secondary health care during adolescence as cases (age 10-17).

### Negative control outcome analyses

There are no established statistical procedures to refine the MR estimate by factoring in the negative control outcome. It has been suggested that calibrating the putative causal estimate with a quantitative contrast between the negative control and the main estimate could lead to bias (for further detail see Sanderson et al. (87)). Therefore, we focused on testing for the degree of confounding rather than refining the MR estimate.

### The smallest effect size of interest

We used the “small telescopes” approach (88) for setting the SESOI, which is particularly suitable for replications. In this approach, the SESOI is set to the effect size that the original study had 33% power to detect. The idea is that based on this power level, the probability of observing an effect (if a true effect exists) is too low to reliably distinguish signal from noise. We calculated the effect size the original study would have 33% power to detect using the *mRnd* power calculator for Mendelian randomisation (89). We used the values from the original study (56) to determine the effect size (*N* = 2,404, *ɑ* = 0.05, *K* = 0.155, *R^2^_XG_* = 0.049) where *N* is the sample size, *ɑ* is the Type-I error rate, *K* is the proportion of cases in the study, and *R^2^_XG_* is the proportion of variance explained for the association between the genetic variants *G_j_* and the exposure *X*. The resulting effect size was *D* = 0.25, which was selected as the SESOI for this analysis. In line with our directional hypothesis and one-tailed null hypothesis significance test, we again applied an inferiority test (setting and testing on an equivalence bound at the SESOI only in the predicted direction).

### Sensitivity analyses

As sensitivity analyses, we used 1) *F*-statistics for the strength of instrument-exposure association; 2) regression of the covariates on the genetic instrument; and 3) a battery of MR sensitivity analyses (including MVMR accounting for BMI).

*Multivariable MR accounting for BMI.* The traditional MR approach assumes that there is no horisontal pleiotropy. We expect that the most likely threat to this assumption is pleiotropy via childhood body size/BMI. Previous studies have attempted to solve this by excluding SNPs associated with childhood (as a proxy for ‘pre-pubertal’) and/or adult (as a proxy for ‘post-pubertal’) BMI (56). However, excluding adult BMI SNPs in particular risks inducing a spurious association with depression, due to collider bias (90). We therefore conducted MVMR analyses with genetic instruments for age at menarche and either childhood body size or adult BMI (this was a deviation due to low conditional instrument strength, see Table 3) included in the same model - estimating the direct effect of age at menarche on the outcome. MVMR accounts for any overlap analytically, whereas excluding SNPs associated with BMI based on *P*-values will likely miss SNPs below the employed threshold. Finally, we also ran a model including the genetic instrument for estradiol to test the direct effect of age at menarche on depressive symptoms when accounting for estradiol.

*MR sensitivity analyses.* In addition, we conducted several sensitivity analyses to assess the three main assumptions of MR: 1) that the instrument is associated with the exposure, 2) that the genetic variants are independent of all confounders, and 3) that the instrument affects the outcome only through the exposure of interest. To evaluate the assumptions, we used 1) *F*-statistics for the instrument-exposure association (where *F* > 10 is required), 2) regression of the covariates on the genetic risk score (statistically significant relationships indicate potential confounding), and 3) MR sensitivity analyses. We excluded SNPs that were associated with more variation in depression than age at menarche. If the causal relationship was then attenuated, this would suggest the existence of other pleiotropic pathways or reverse causation. Also, we conducted multiple two-sample sensitivity analyses (MR-Egger, MR-PRESSO, weighted median and contamination mixture). The MR-Egger intercept and MR-PRESSO global test were used to test for bias from directional horizontal pleiotropy. For MR-Egger, a significant intercept would be considered indicative of bias from directional horizontal pleiotropy. For MR-PRESSO, we would report the outlier-corrected causal estimate if both the global test and the distortion test were significant. Note that sensitivity analyses such as MR-Egger are subject to their own biases, and therefore the strongest indication that results are unlikely to be biased by horisontal pleiotropy would be consistent evidence across the different methods.

*Dichotomised outcome.* We also ran the same analyses with a dichotomised version of the SMFQ as the outcome, answering whether we could replicate the result in Sequeira et al. (56).

### Inference criteria

*H3a.* Support for hypothesis 3a was assessed based on whether or not 1) the coefficient for the causal effect of age at menarche on 14-year depressive symptoms was significantly less than zero (one-tailed test; alpha 5%); 2) we failed to reject the null hypothesis that this causal effect in the population was at least as large as the SESOI (one-tailed test; alpha 5%); and 3) we failed to reject the null hypothesis that this causal effect in the population was at least as large as the lower bound of the negative control (8-year) estimate (one-tailed test; alpha 5%).

*H3b.* Support for hypothesis 3b was assessed based on whether 1) the coefficient for the causal effect of age at menarche on depressive disorders was significantly less than zero (one-tailed test; alpha 5%); and 2) we failed to reject the null hypothesis that this causal effect in the population was at least as large as the SESOI (one-tailed test; alpha 5%).

## Question 4: Is there evidence of causal links between age at menarche and other domains of mental health?

### Main analyses

*H4.1-4a.* We hypothesised that age at menarche would causally affect symptoms in other domains: anxiety (H4.1a); CD (H4.2a); ODD (H4.3a); and ADHD (H4.4a). To test each of these hypotheses, we used 2SLS to estimate the effect of age at menarche on each symptom domain at age 14. We also ran negative control analyses using the corresponding symptom domains at age 8 as negative control outcomes. We conducted the same two-sample MR sensitivity analyses as in H3a. In addition, to mirror the observational analyses accounting for overlap with depression, we included an instrument for depression alongside age at menarche in an MVMR analysis.

*H4.1-3b.* We hypothesised that the genetic risk score for age at menarche would be associated with diagnoses in other domains: anxiety disorders (H4.1b); DBD, including CD and ODD (H4.2b); and ADHD (H4.3b). To test each of these hypotheses, we used 2SLS regression to test the relationship between the genetic risk score for age at menarche and diagnoses of each condition. These analyses included any relevant diagnosis in either primary or secondary health care during adolescence (age 10-17).

### The smallest effect size of interest

For these analyses, there were no prior MR studies to base our estimated effect size on. Therefore, our SESOI was set to *D* = 0.20 (i.e., what is considered a small effect size, in the absence of a clear theoretical justification). Thus, the equivalence bounds were -0.20 - 0.20.

### Inference criteria

*H4.1-4a.* Support for hypotheses 4.1-4a was assessed based on whether or not 1) the coefficient for the causal effect of age at menarche on a domain of 14-year symptoms was different from zero in the fully adjusted model (two-tailed tests, 5% alpha); 2) we failed to reject the null hypothesis that the causal effect in the population was at least as extreme as the SESOI in either direction (two one-tailed tests, 5% alpha); and 3) we failed to reject the null hypothesis that the causal effect in the population was at least as large as the lower bound of the negative control (8-year) estimate (one-tailed test; alpha 5%).

*H4.1-3b.* Support for hypotheses 4.1-3b was assessed based on whether or not 1) the coefficient for the causal effect of age at menarche on a diagnosis was different from zero (two-tailed tests, 5% alpha); and 2) we failed to reject the null hypothesis that the causal effect in the population was at least as extreme as the SESOI in either direction (two one-tailed tests, 5% alpha).
