# Supplementary material for: Assessing causal links between age at menarche and adolescent mental health: a Mendelian randomisation study
Source: BMC Med. 2024 Apr 12;22:155. doi: 10.1186/s12916-024-03361-8 (PMC11015655; doi:10.1186/s12916-024-03361-8)
Supplement: Supplementary file 7 — Additional file 7. Description of power analyses, including: a) projected prevalence, b) data generation, c) power calculation, d) Figs. S1-S8 showing results of power analysis for all hypotheses. [file 12916_2024_3361_MOESM7_ESM.docx]

## Additional file 7: Power analyses

### Projected prevalence

In the Stage 1 submission we estimated the expected prevalence of diagnostic outcomes, derived from registry data, at the anticipated time of analysis and in the analytic sample (14-year questionnaire returners with and without genetic data). To do this we calculated rates for all relevant diagnoses among female MoBa participants who already have registry follow-up for all years between the ages of 10 and 17 (inclusive). We projected these onto 100 random samples (50 with *N* = 12,500 and 50 with *N* = 10,000) of girls whose mothers responded to the previous MoBa questionnaire. This assumed imputation of diagnoses to account for censoring, which we did not do (see Table 3). The projected prevalence rates were 13.5% for depression, 16.1% for anxiety, 2.5% for CD/ODD, and 13.9% for ADHD.

### Data generation

For all power analyses, we simulated 1000 replicate datasets under various possible scenarios of data availability and effect sizes. For analyses with diagnoses as outcomes, we also varied case rates within plausible ranges. For all replicates across all scenarios and analyses, we began by simulating an age at menarche variable, sampling from a normal distribution with a mean of 151.52 (months) and standard deviation of 14.11 (values from Sequeira et al. (1)), rounded to the nearest year (to replicate the response format of the age at menarche item in the MoBa 14-year questionnaire). The simulated variable was allowed to take values > 14 years, meaning that all power analyses assume imputation of this variable (see Additional file 1) has already taken place. Details of the other variables simulated in the power analyses for each hypothesis are given below.

### Power calculation

Power for NHSTs (detailed below) was calculated empirically as the proportion of replicates in each scenario in which the null hypothesis was rejected with a 5% alpha. Power for equivalence tests was calculated empirically as the proportion of replicates in each scenario in which the null hypothesis of the equivalence test (i.e., in the case of a two-tailed test, that an effect is not equivalent to zero; and in the case of a one-tailed test, that an effect is not smaller than the SESOI) was rejected with a 5% alpha.

*Hypotheses 1a/b.* For H1a we additionally simulated a depressive symptoms variable (*M* = 5.71, *SD* = 4.93; values from Sequeira et al., with a floor effect at 0. In the range of scenarios simulated for this hypothesis, the correlation between age at menarche and depressive symptoms was specified at each *r* from 0 to -0.15 in increments of 0.01, for sample sizes of 12,000 and 13,000 respectively. Results indicated 95% power to detect an effect of Cohen’s *D* ≥ 0.08 at *N* = 12,000 and ≥ 0.06 at *N* = 13,000 (full results for all scenarios are presented in Figure S1 below). For H1b we additionally simulated a binary depression diagnosis variable, based on a pre-specified prevalence and association with age at menarche (varied across scenarios at, respectively, 2%, 6%, 10%, 14% and *D* = 0 to -0.26 in increments of 0.02). Results indicated 95% power to detect an effect of Cohen’s *D* ≥ 0.14 at both *N* = 12,000 and *N* = 13,000 for depression prevalence of 6% or higher (full results for all scenarios are presented in Figure S2).

*Hypotheses 2.1-4a/2.1-3b.* For H2.1-4a we additionally simulated anxiety, CD, ODD, and ADHD symptoms, with distributions based on the same variables in MoBa data at 8 years. As for hypothesis 1a, we simulated data for scenarios with age at menarche-outcome correlations specified from 0 to -0.15 in increments of 0.01, for sample sizes of 12,000 and 13,000 respectively. Results indicated 95% power to detect an effect of Cohen’s *D* ≥ 0.12 at both simulated sample sizes (full results for all scenarios are presented in Figure S3). For H2.1-3b, the simulation was essentially identical to H1b (with different equivalence bounds and use of two-tailed tests - see analysis plan for details). Results indicated 95% power to detect an effect of Cohen’s *D* ≥ 0.20 at *N* = 12,000 and ≥ 0.18 at *N* = 13,000 for diagnosis prevalence of 2% or higher (full results for all scenarios are presented in Figure S4).

*Hypotheses 3a/b.* For H3a we additionally simulated a genetic instrument for age at menarche (*M* = 0, *SD* = 1), and a depressive symptoms variable as per H1a. The *R^2^* of the genetic instrument for the simulated age at menarche variable was set at 0.05, 0.075, and 0.10 across scenarios. The association between simulated age at menarche and the depressive symptoms variable was based on the average causal effect (specified from *D* = 0 to -0.30 in increments of -0.01 across scenarios) plus an observational confounding effect (drawn randomly from a normal distribution *M* = 0, *SD* = 0.05 on the *D* scale for each replicate). Results indicated 95% power to detect an average causal effect (using 2SLS) of Cohen’s *D* ≥ 0.2 when the R^2^ of the instrument is 0.075 or above at either simulated sample size (full results for all scenarios are presented in Figure S5). For H3b we again simulated the genetic instrument for age at menarche at *R^2^* 0.05, 0.075, and 0.10 across scenarios. As per H1b, a depressive diagnosis outcome was simulated with prevalence ranging from 0.02 to 0.14 across scenarios, with its relationship to simulated age at menarche parameterised as the causal odds (specified from *D* = 0 to -0.30 in increments of -0.02 across scenarios) plus an observational confounding effect as in H3a above. Results indicated 95% power to detect a causal effect (using logistic 2SLS with robust standard errors) of Cohen’s *D* ≥ 0.24 when the R^2^ of the instrument is ≥ 0.075 and depression prevalence 14% at either simulated sample size (full results for all scenarios are presented in Figure S6).

*Hypotheses 4.1-4a/4.1-3b.* Simulations for H4.1-4a were essentially identical to those for H3a (with different equivalence bounds and use of two-tailed tests - see *Analysis plan* for details). Results indicated 95% power to detect an average causal effect (using 2SLS) of Cohen’s *D* ≥ 0.2 when the R^2^ of the instrument is 0.10 at either simulated sample size and 80% power to detect Cohen’s *D* ≥ 0.2 with the R^2^ of the instrument at ≥ 0.075 (full results for all scenarios are presented in Figure S7). Simulations for H4.1-3b were essentially identical to those for H3b (with different equivalence bounds and use of two-tailed tests - see Analysis plan for details). Results indicated 95% power to detect a causal effect (using logistic 2SLS with robust standard errors) of Cohen’s *D* ≥ 0.26 when the R^2^ of the instrument is ≥ 0.075 and diagnosis prevalence is 14% in either simulated sample size and 80% power for Cohen’s *D* ≥ 0.20 in the same scenarios (full results for all scenarios are presented in Figure S8).

##
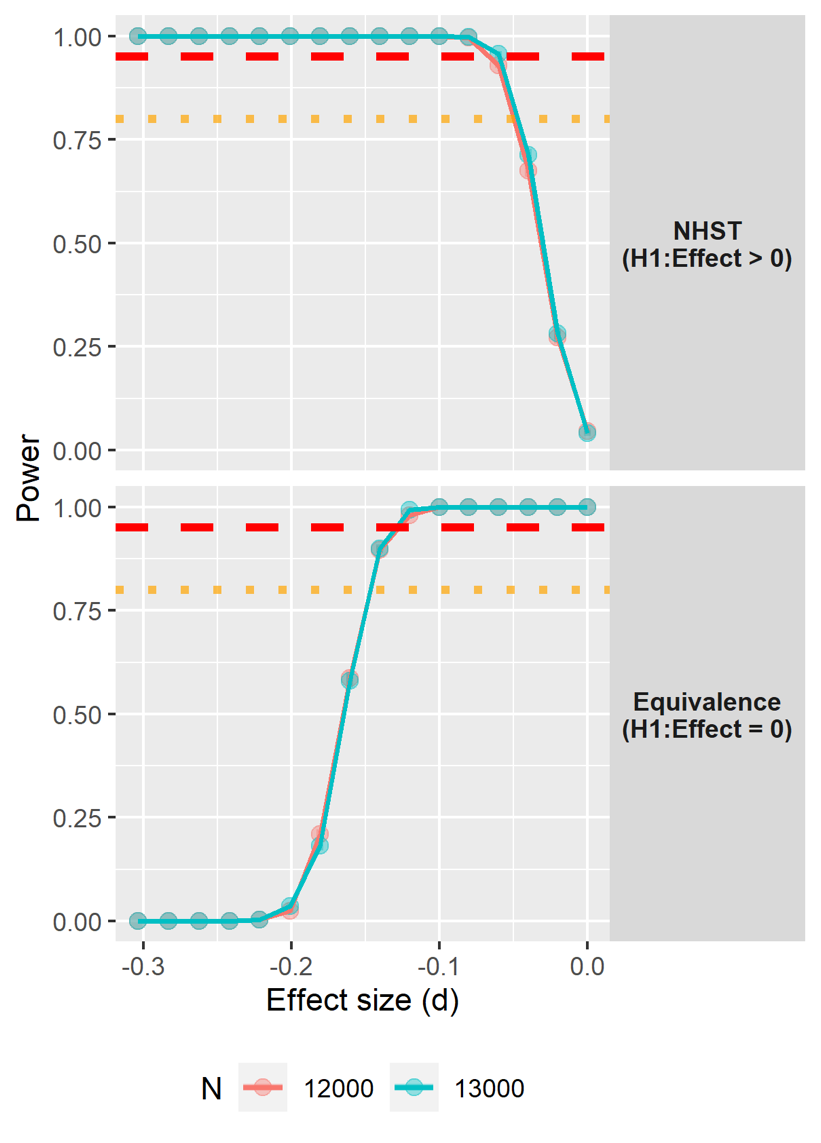


## Figure S1. Power for NHST and equivalence test for H1a. Power for testing hypothesis 1a at a range of potential effect sizes, with N=12,000 (light red) or N=13,000 (light green); dashed red line is the 95% power threshold, dotted yellow line is the 80% power threshold; NHST, null hypothesis significance testing.

**
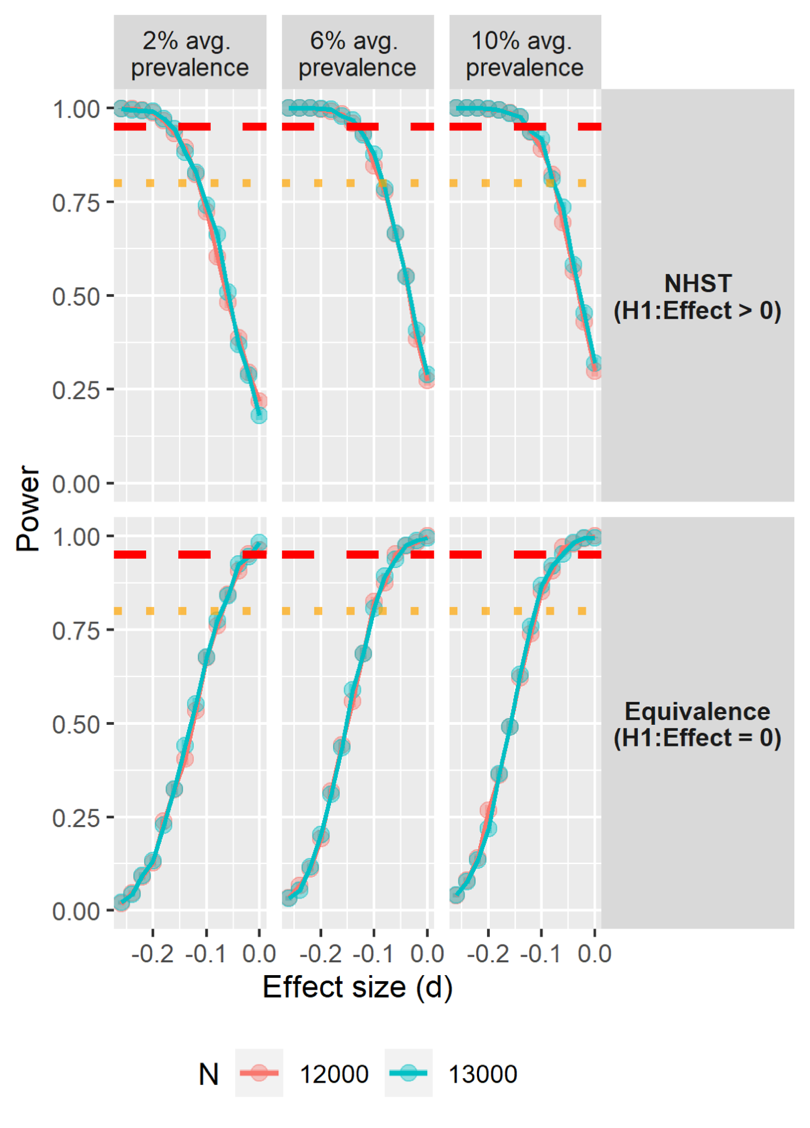
**
**Figure S2. Power for NHST and equivalence test for H1b.**Power for testing hypothesis 1b at a range of potential effect sizes, with N=12,000
(light red) or N=13,000 (light green); dashed red line is the 95% power threshold,
dotted yellow line is the 80% power threshold; NHST, null hypothesis significance
testing.


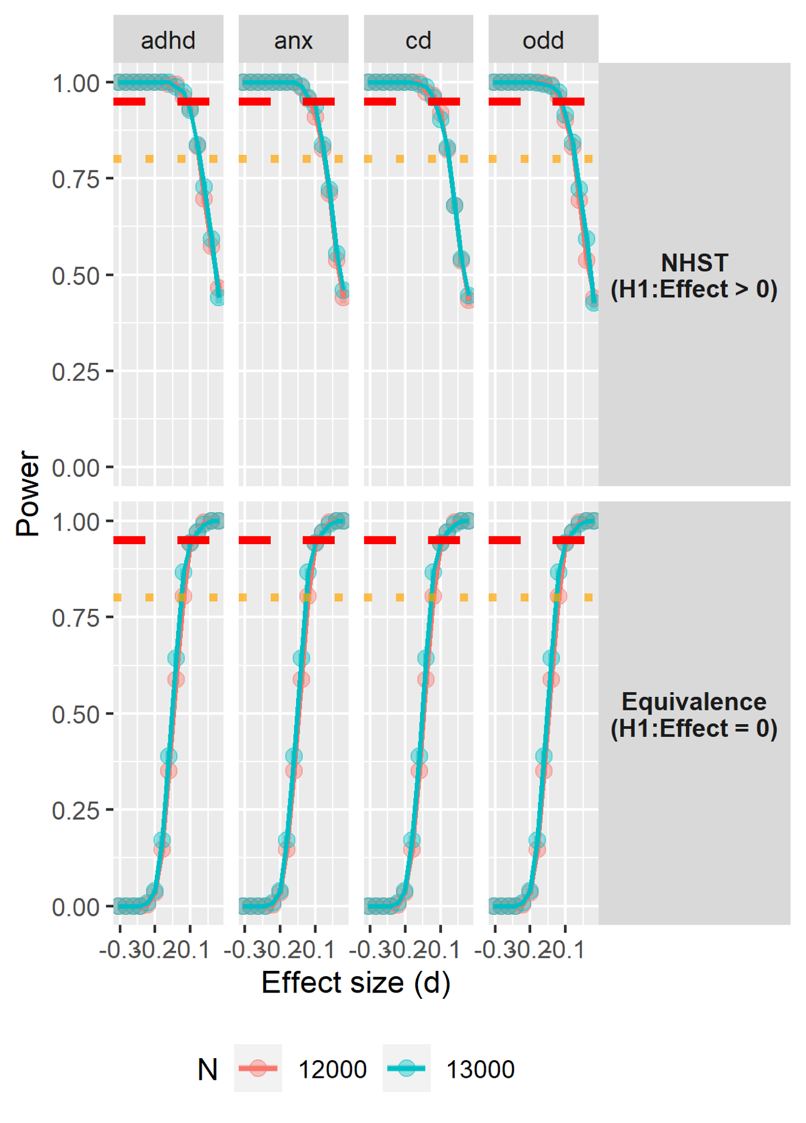

**Figure S3. Power for NHST and equivalence test for H2a.**Power for testing hypothesis 2a at a range of effect sizes for each symptom domain, with
N=12,000 (light red) or N=13,000 (light green); dashed red line is the 95% power threshold,
dotted yellow line is the 80% power threshold; adhd, attention-deficit hyperactivity disorder;
anx, anxiety; cd, conduct disorder; odd, oppositional defiant disorder; NHST, null hypothesis significance testing.


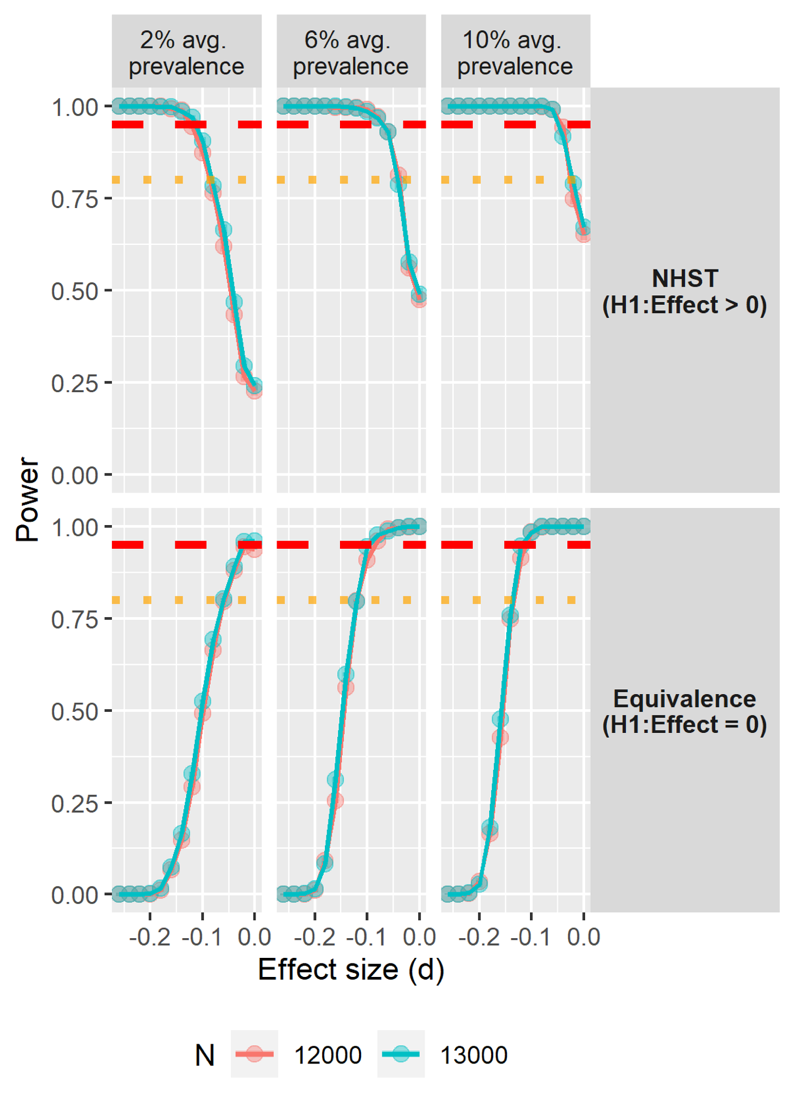
 **Figure S4. Power for NHST and equivalence test for H2b.**Power for testing hypothesis 2b at a range of effect sizes for 2%, 6%, and 10% avg.
prevalence of a disorder, with N=12,000 (light red) or N=13,000 (light green); dashed
red line is the 95% power threshold, dotted yellow line is the 80% power threshold;
NHST, null hypothesis significance testing.

##


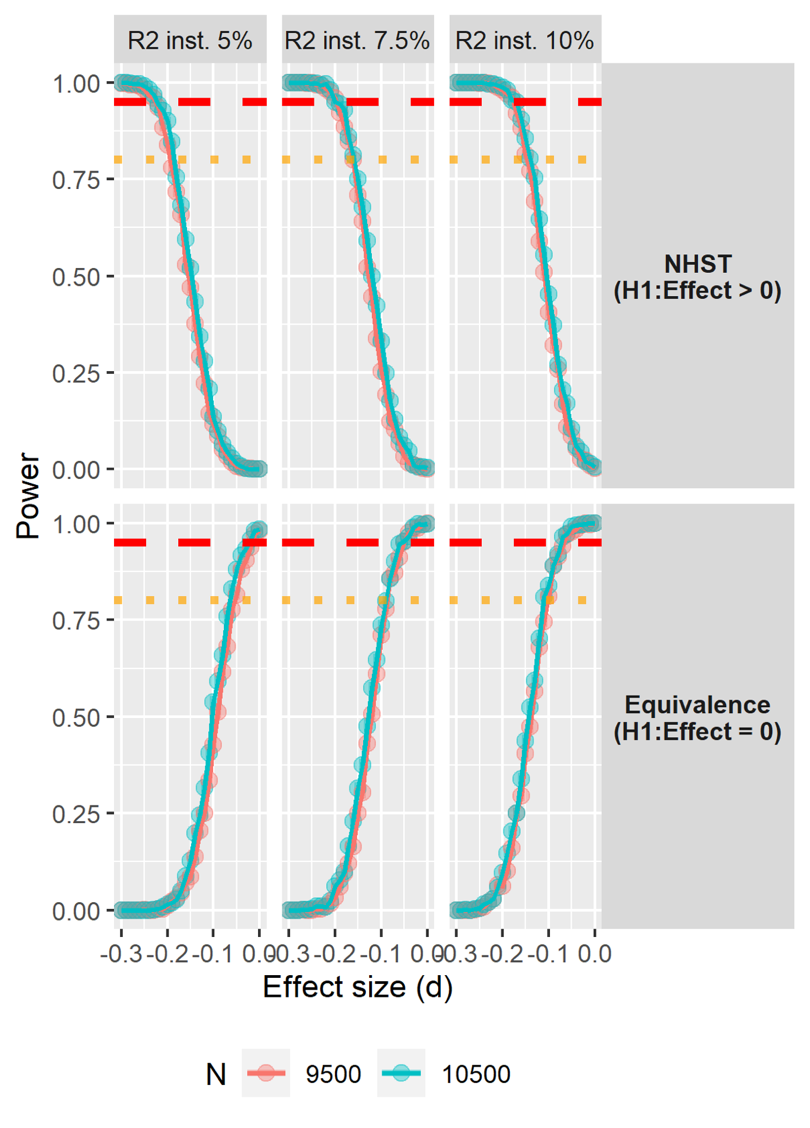

**Figure S5. Power for NHST and equivalence test for H3a.**Power for testing hypothesis 3a at a range of effect sizes with 5%, 7.5%, and 10%
instrument strength, at N=9,500 (light red) or N=10,500 (light green); dashed red
line is the 95% power threshold, dotted yellow line is the 80% power threshold; R2
inst, R^2^ instrument-exposure association; NHST, null hypothesis significance testing.

##

## *^
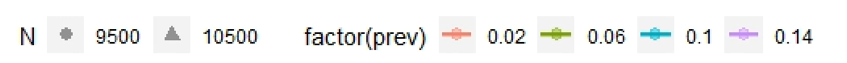
^*
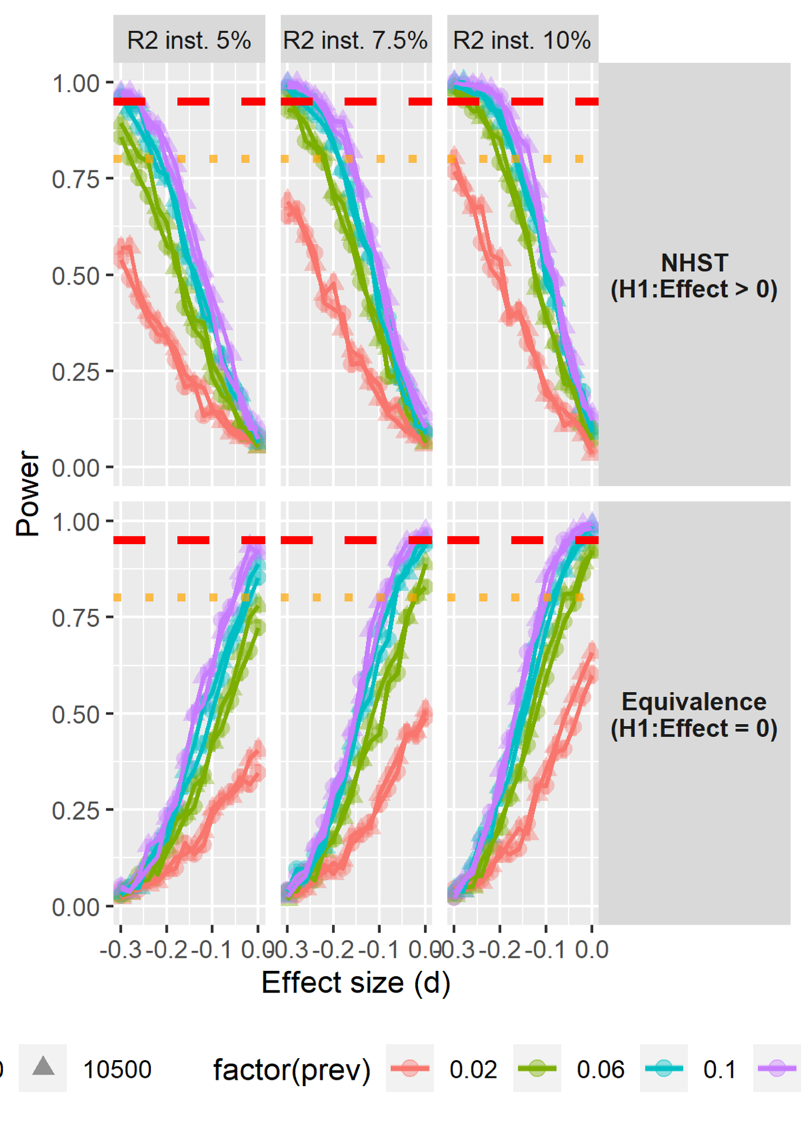
 Figure S6. Power for NHST and equivalence test for H3b. Power for testing hypothesis 3b at a range of effect sizes with 5%, 7.5%, and 10% instrument strength, across four prevalence rates (2% in light red, 6% in green, 10% in light blue, and 14% in purple), at N=9,500 (points) or N=10,500 (triangles); dashed red line is the 95% power threshold, dotted yellow line is the 80% power threshold; R2 inst, R^2^ instrument-exposure association; NHST, null hypothesis significance testing.


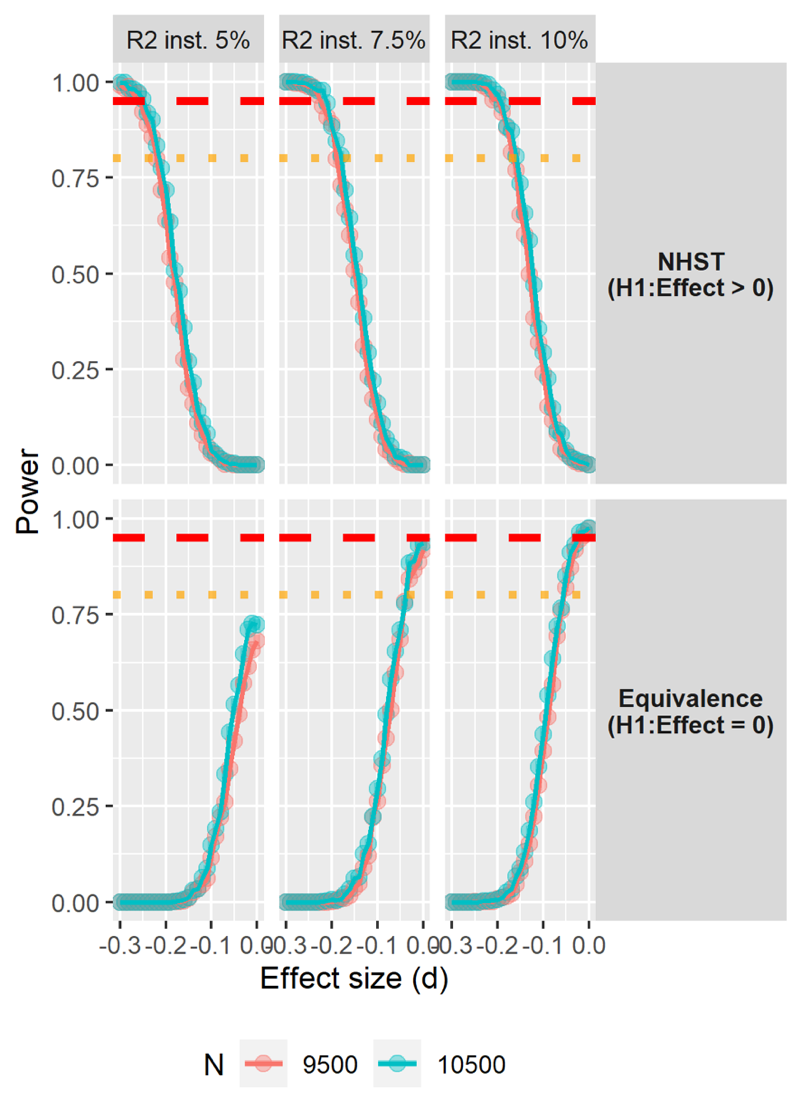

**Figure S7. Power for NHST and equivalence test for H4a.**Power for testing hypothesis 4a at a range of effect sizes with 5%, 7.5%, and 10%

## instrument strength, with N=9,500 (light red) or N=10,500 (light green); dashed red

## line is the 95% power threshold, dotted yellow line is the 80% power threshold; R^2^

## inst, R^2^ instrument-exposure association; NHST, null hypothesis significance testing.

*^
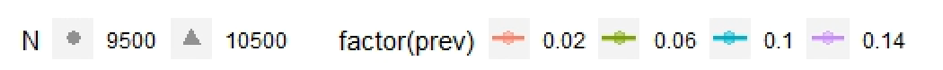
^*
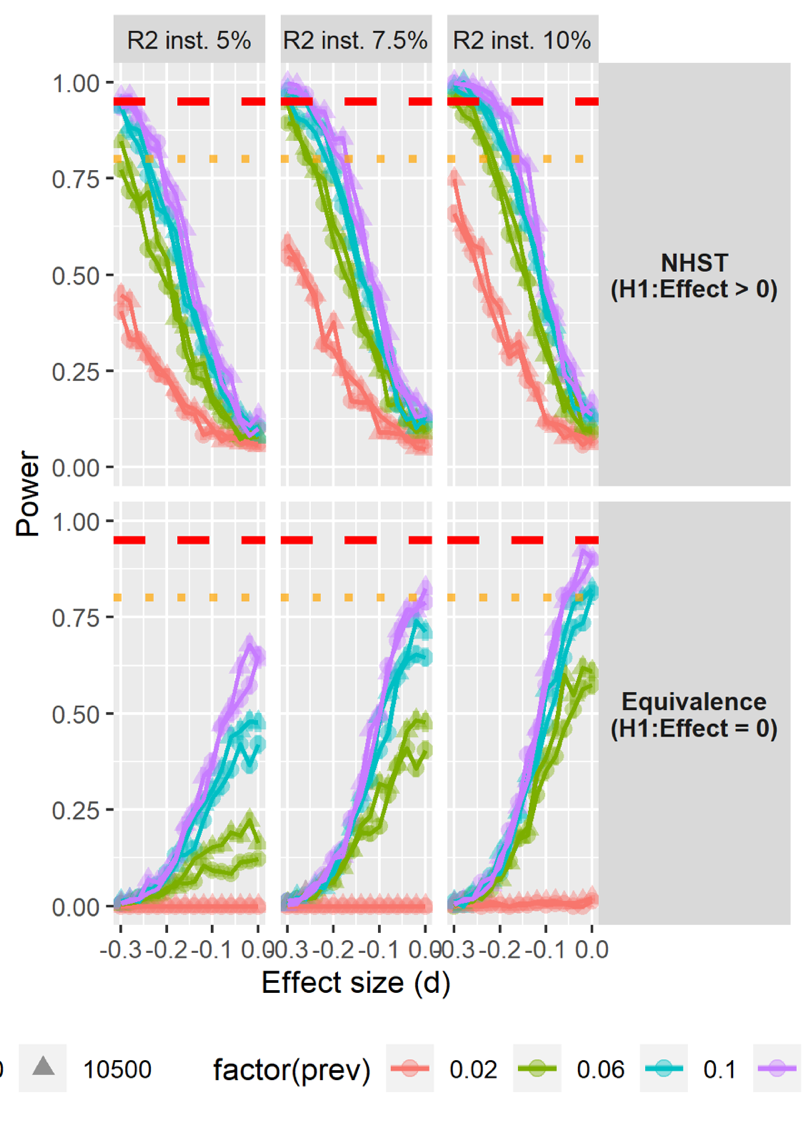
 **Figure S8. Power for NHST and equivalence test for H4b.**Power for testing hypothesis 4b at a range of effect sizes with 5%, 7.5%, and 10%
instrument strength, across four prevalence rates (2% in light red, 6% in green, 10%
in light blue, and 14% in purple), at N=9,500 (points) or N=10,500 (triangles); dashed
red line is the 95% power threshold, dotted yellow line is the 80% power threshold; R2
inst, R^2^ instrument-exposure association; NHST, null hypothesis significance testing.
