# Supplementary material for: Assessing causal links between age at menarche and adolescent mental health: a Mendelian randomisation study
Source: BMC Med. 2024 Apr 12;22:155. doi: 10.1186/s12916-024-03361-8 (PMC11015655; doi:10.1186/s12916-024-03361-8)
Supplement: Supplementary file 8 — Additional file 8. Supplementary results, including: a) imputation of age at menarche, b) breast stage as an additional exposure, c) categorised age at menarche, d) exploratory analyses. [file 12916_2024_3361_MOESM8_ESM.docx]

## Additional file 8: Supplementary results

### Imputation of age at menarche

# We conducted a sensitivity analysis testing the accuracy of the imputation of age at menarche. This involved setting 972 random age at menarche values to missing, before imputing these values. In this analysis, the mean age at menarche was 12.55 for the actual values, and 12.61 for the imputed values. The mean absolute error was 0.73 and the correlation between actual and imputed values was 0.46.

### Breast stage as an additional exposure

A more advanced breast stage was associated with more depression diagnoses (OR = 1.36, 95% CI [1.17, 1.60], p < 0.01) but not depressive symptoms (β = 0.01, 95% CI [-0.02, 0.05], p = 0.37). Including both exposures in the same models did not attenuate the associations of age at menarche with either depression diagnoses (OR = 0.80, 95% CI [0.75, 0.85], p_one-tailed_ < 0.01) or depressive symptoms (β = -0.11, 95% CI [-0.13, -0.10], p_one-tailed_ < 0.01).

### Categorised age at menarche

To facilitate comparison with previous studies, we also conducted observational analyses based on age at menarche categorised into ‘early’ (n = 1,832), ‘average’ (n = 8,338), and ‘late’ (n = 3,228), showing linear associations with dichotomised depressive symptoms and depression diagnoses (linear trends p < 0.01). Compared to ‘average’ menarche, an ‘early’ menarche was associated with higher prevalence of depression (symptoms: OR = 1.27, 95% CI [1.12, 1.44], p_one-tailed_ < 0.01; diagnoses: OR = 1.47, 95% CI [1.20, 1.80], p_one-tailed_ < 0.01), and ‘late’ menarche was associated with lower prevalence (symptoms: OR = 0.75, 95% CI [0.66, 0.85], p_one-tailed_ < 0.01; diagnoses: OR = 0.69, 95% CI [0.55, 0.86], p_one-tailed_ < 0.01).

### Exploratory analyses

We conducted MR analyses separately for new ADHD diagnoses in preadolescence (age 9-11; N = 111), early adolescence (age 12-14; N = 98), and mid-late adolescence (age 15-17; N = 123). Results showed no effect in preadolescence (OR = 1.03, 95% CI [0.46, 2.27]). Notably, the effect on ADHD diagnoses was stronger in early (OR = 0.47, 95% CI [0.23, 0.95]) than mid-late adolescence (OR = 0.83, 95% CI [0.44, 1.56]). A similar pattern emerged for depression diagnoses (Ns = 29 pre, 226 early, and 439 mid-late adolescence), where there was evidence of a causal effect in early (OR = 0.50, 95% CI [0.26, 0.95]) but not mid-late adolescence (OR = 0.95, 95% CI [0.64, 1.41]). Finally, we conducted a similar analysis for new anxiety diagnoses (Ns = 181 pre, 383 early, and 512 mid-late adolescence), where the pattern of findings was different. Results showed no effects in preadolescence (OR = 1.12, 95% CI [0.60, 2.10]), early adolescence (OR = 0.85, 95% CI [0.56, 1.29]), or mid-late adolescence (OR = 0.75, 95% CI [0.52, 1.08]). There were too few DBD cases to subdivide them into different time windows.
