# Supplementary material for: Assessing causal links between age at menarche and adolescent mental health: a Mendelian randomisation study
Source: BMC Med. 2024 Apr 12;22:155. doi: 10.1186/s12916-024-03361-8 (PMC11015655; doi:10.1186/s12916-024-03361-8)
Supplement: Supplementary file 9 — Additional file 9: Table S3. With associations of genetic instrument for age at menarche with the covariates. [file 12916_2024_3361_MOESM9_ESM.docx]

**Table S3. Associations of genetic instrument for age at menarche with covariates.**

| **Covariate** | **Estimate** | **Lower 95% CI** | **Upper 95% CI** | **P(>\|z\|)** |
| --- | --- | --- | --- | --- |
| Parental education | 0.01 | -0.01 | 0.03 | 0.31 |
| Parental income | 0.02 | -0.01 | 0.04 | 0.14 |
| Parental cohabitation 18 months | 0.00 | -0.02 | 0.03 | 0.74 |
| Parental cohabitation 3 years | 0.01 | -0.03 | 0.02 | 0.68 |
| Paternal age | 0.00 | -0.01 | 0.02 | 0.63 |
| Maternal age | 0.02 | 0.00 | 0.04 | 0.02 |
| Financial problems | -0.01 | -0.03 | 0.02 | 0.57 |
| Maternal depression 17 weeks | -0.01 | -0.03 | 0.01 | 0.28 |
| Maternal depression 30 weeks | 0.00 | -0.02 | 0.03 | 0.63 |
| Maternal postnatal depression 6 months | -0.01 | -0.03 | 0.01 | 0.51 |
| Parity | 0.00 | -0.02 | 0.02 | 0.97 |
| BMI 8 years | -0.08 | -0.10 | -0.05 | <0.01 |
| BMI 14 years | -0.10 | -0.12 | -0.08 | <0.01 |
| Child age at questionnaire return 8 years | 0.00 | -0.02 | 0.02 | 0.95 |
| Child age at questionnaire return 14 years | 0.00 | -0.02 | 0.02 | 0.81 |

standardised results of linear regressions with the genetic instrument for age at menarche predicting each covariate; BMI, body mass index; CI, confidence interval.
