# Supplementary material for: Assessing causal links between age at menarche and adolescent mental health: a Mendelian randomisation study
Source: BMC Med. 2024 Apr 12;22:155. doi: 10.1186/s12916-024-03361-8 (PMC11015655; doi:10.1186/s12916-024-03361-8)
Supplement: Supplementary file 10 — Additional file 10: Table S4. With results of multivariable Mendelian randomisation sensitivity analyses. [file 12916_2024_3361_MOESM10_ESM.docx]

**Additional file 10: MVMR sensitivity results**

**Table S4.** Results of multivariable mendelian randomisation (MVMR) sensitivity analyses.

| **Additional exposure** | **Outcome** | **MVMR-Egger** | | | **MVMR-Median** | | | **MVMR-Lasso** | | |
| --- | --- | --- | --- | --- | --- | --- | --- | --- | --- | --- |
|  |  | **EST** | **LCI** | **UCI** | **EST** | **LCI** | **UCI** | **EST** | **LCI** | **UCI** |
| Childhood body size | Depressive sx | 0.01 | -0.06 | 0.08 | -0.03 | -0.10 | 0.03 | -0.03 | -0.07 | 0.01 |
| Adult BMI | Depressive sx | 0.03 | -0.04 | 0.09 | -0.00 | -0.07 | 0.06 | -0.01 | -0.05 | 0.04 |
| Estradiol | Depressive sx | 0.02 | -0.06 | 0.09 | -0.06 | -0.12 | 0.00 | -0.04 | -0.08 | -0.00 |
| Major depression | Anxiety sx | -0.02 | -0.08 | 0.05 | -0.05 | -0.11 | 0.01 | -0.01 | -0.05 | 0.03 |
| Major depression | Conduct sx | 0.01 | -0.05 | 0.07 | -0.02 | -0.08 | 0.04 | -0.02 | -0.26 | 0.21 |
| Major depression | Oppositional sx | 0.01 | -0.05 | 0.08 | -0.01 | -0.07 | 0.05 | 0.00 | -0.04 | 0.04 |
| Major depression | ADHD traits | 0.02 | -0.04 | 0.08 | -0.01 | -0.07 | 0.06 | 0.01 | -0.03 | 0.04 |

sx, symptoms; EST, estimate; LCI, lower confidence interval; UCI, upper confidence interval; 95% confidence intervals are presented for all outcomes.
