# Supplementary material for: Assessing causal links between age at menarche and adolescent mental health: a Mendelian randomisation study
Source: BMC Med. 2024 Apr 12;22:155. doi: 10.1186/s12916-024-03361-8 (PMC11015655; doi:10.1186/s12916-024-03361-8)
Supplement: Supplementary file 11 — Additional file 11: Fig. S9. With differences between participants and non-participants with IP weighting. [file 12916_2024_3361_MOESM11_ESM.docx]

**Additional file 11: Inverse probability weights**


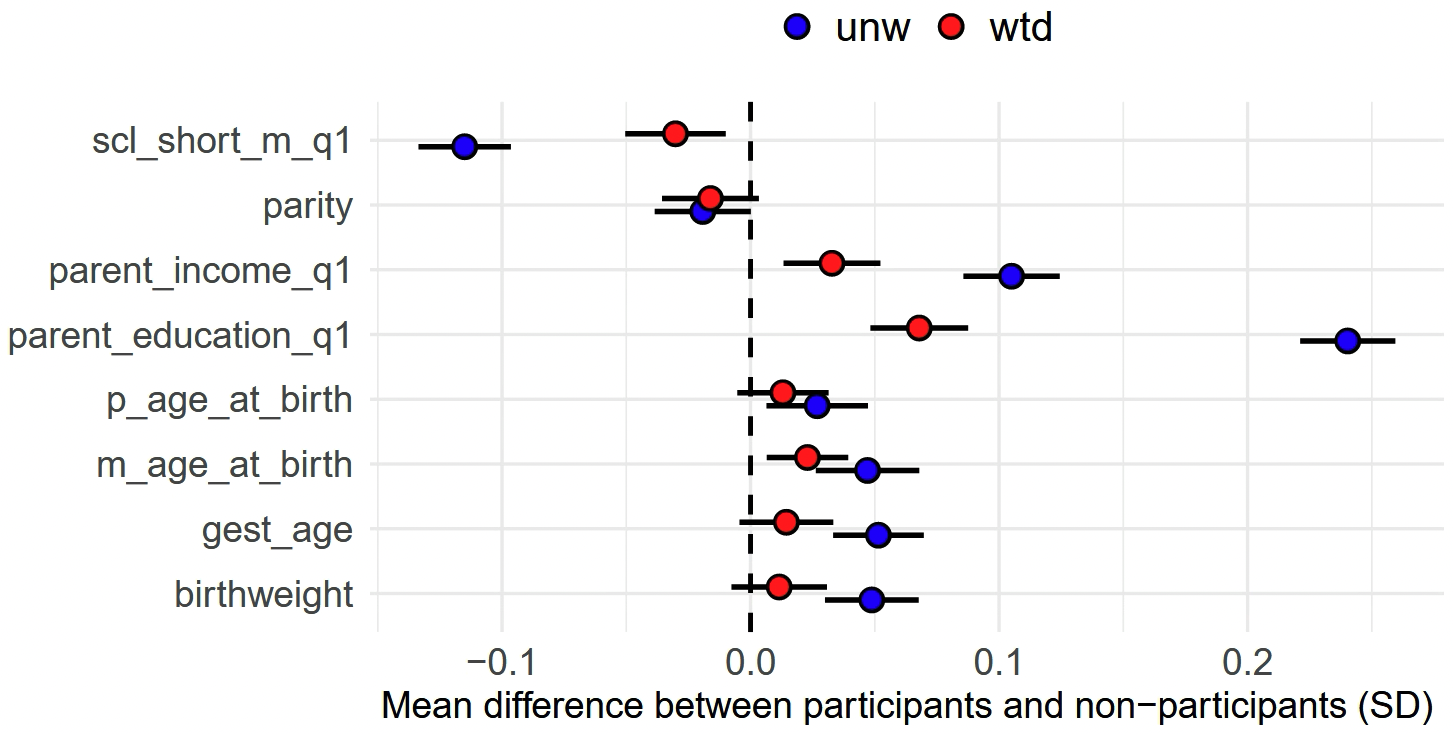

**Figure S9. Differences between participants and non-participants with weighting.**Mean differences in the level of continuous covariates before (“unw”) and after (“wtd”) inverse probability weighting. Gestational age (“gest_age”) and birthweight were added to the model in
order to improve the prediction.
