# Supplementary material for: Assessing causal links between age at menarche and adolescent mental health: a Mendelian randomisation study
Source: BMC Med. 2024 Apr 12;22:155. doi: 10.1186/s12916-024-03361-8 (PMC11015655; doi:10.1186/s12916-024-03361-8)
Supplement: Supplementary file 12 — Additional file 12: Fig. S10. Showing the impact of inverse probability weighting on results for depression. [file 12916_2024_3361_MOESM12_ESM.docx]

**Additional file 12: Impact of inverse probability weighting**

**
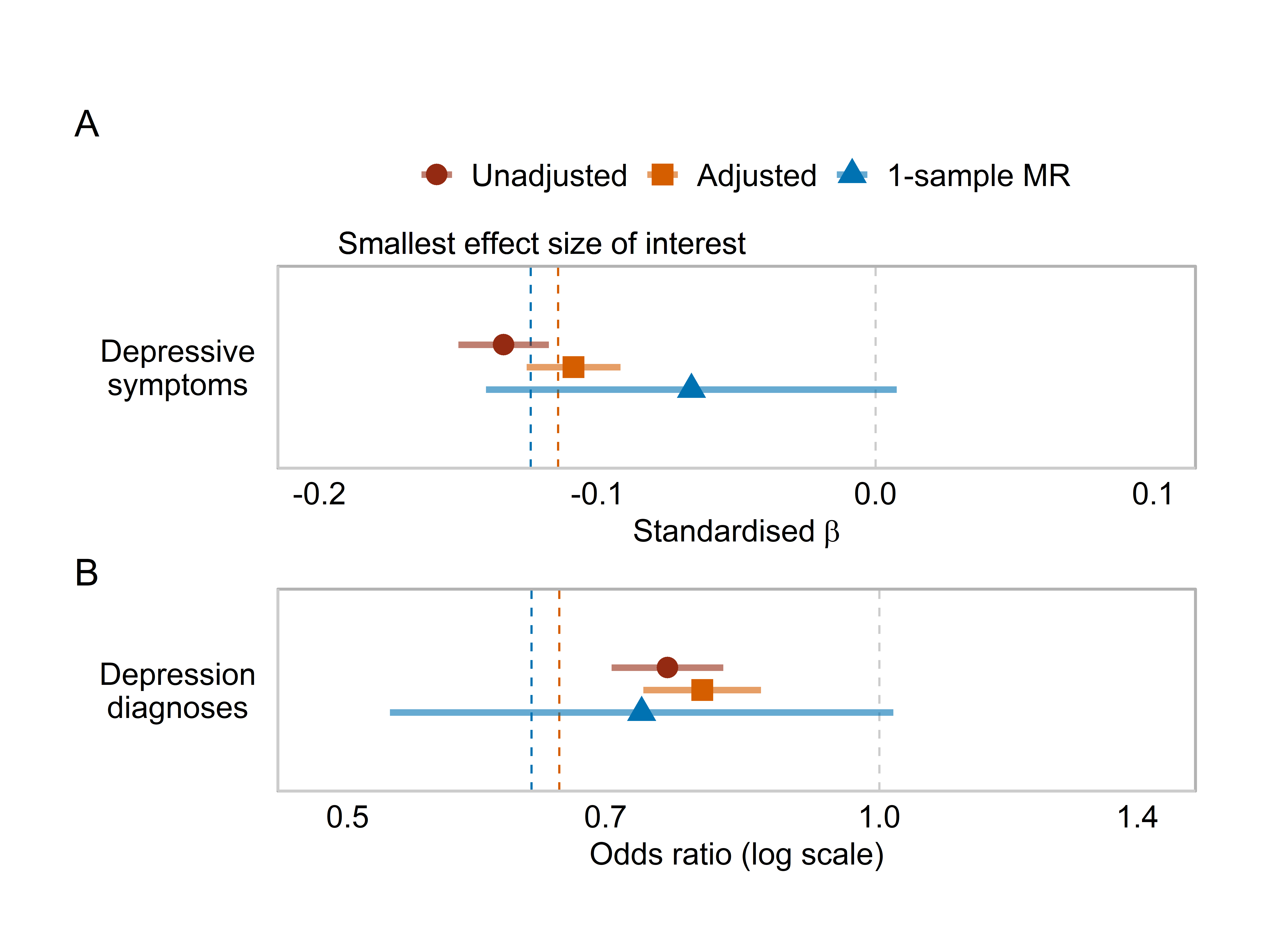

Figure S10. Age at menarche and depression links after inverse probability weighting.**The figure shows minimal differences in point estimates for **A**) symptoms and **B**) diagnoses of depression, as compared to the estimates in the main manuscript, after inverse probability weighting (beyond reduced precision, which is due to the incorporation of weights in the model). This seems to suggest little impact of selective attrition on the results.
