# Supplementary material for: Assessing causal links between age at menarche and adolescent mental health: a Mendelian randomisation study
Source: BMC Med. 2024 Apr 12;22:155. doi: 10.1186/s12916-024-03361-8 (PMC11015655; doi:10.1186/s12916-024-03361-8)
Supplement: Supplementary file 13 — Additional file 13: Fig. S11. With 2-sample MR sensitivity analyses for other mental health domains. [file 12916_2024_3361_MOESM13_ESM.docx]

**Additional file 13: 2-sample MR sensitivity analyses other domains**

**
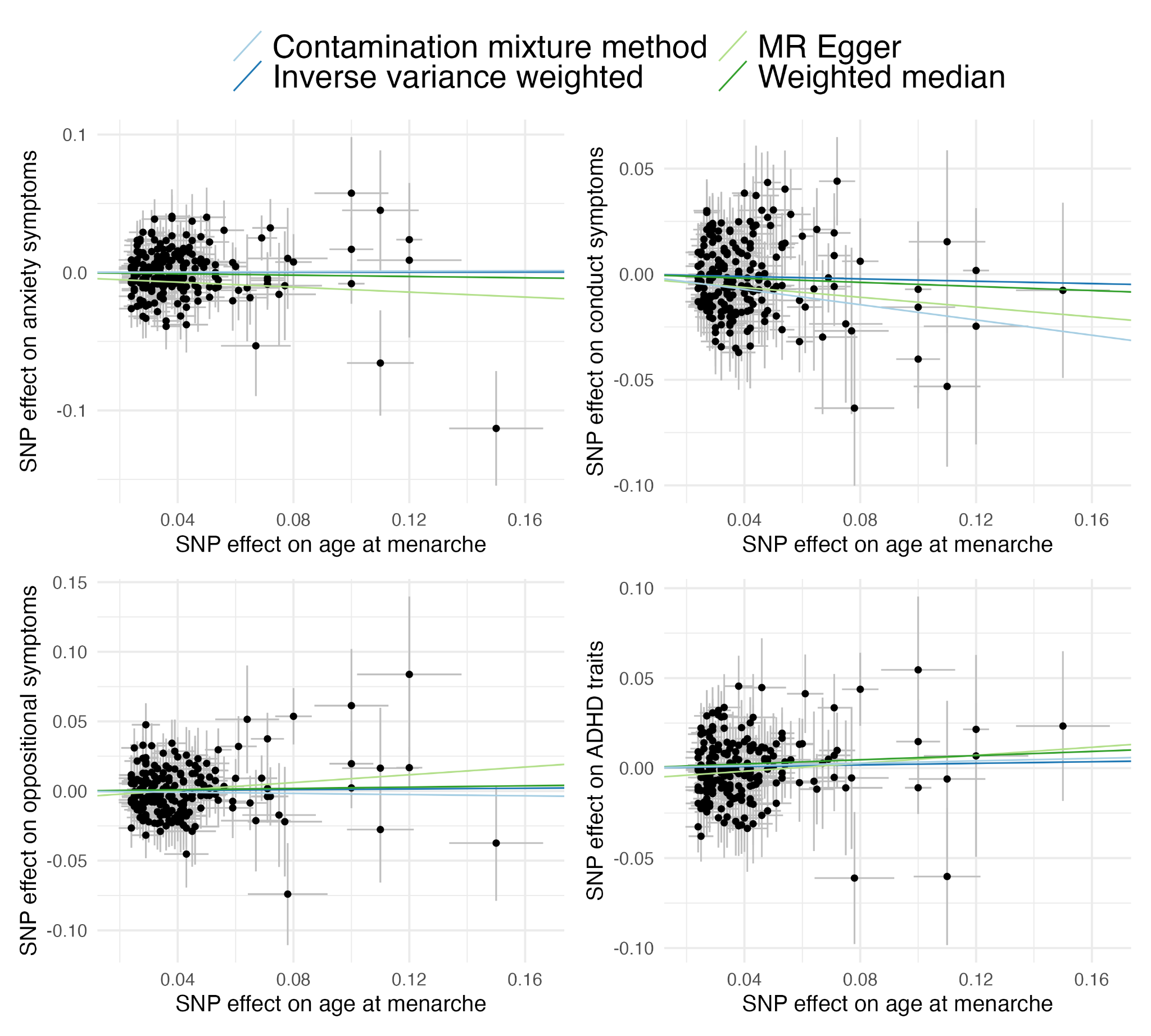

Figure S11. 2-sample MR sensitivity analyses of age at menarche and other domains.** MR sensitivity analyses showing results that are broadly consistent with the 1-sample MR, with little support for causal relationships between age at menarche and other symptom domains, apart from conduct disorder symptoms. MR, Mendelian randomisation; SNP, single nucleotide polymorphism.
